# Pharmacological Targeting of Angiogenesis in Head and Neck Cancer: Molecular Mechanisms and Emerging Therapeutic Strategies

**DOI:** 10.3390/ph19060950

**Published:** 2026-06-18

**Authors:** Diana Szekely, Antonia Armega-Anghelescu, Alina Cristina Barb, Dorin Novacescu, Catalin Dumitru, Alexia Manole, Radu Gheorghe Dan, Flavia Zara

**Affiliations:** 1 Doctoral School, “Victor Babes” University of Medicine and Pharmacy Timisoara, E. Murgu Square, No. 2, 300041 Timisoara, Romania; diana.szekely@umft.ro; 2 Department II of Microscopic Morphology, Discipline of Histology, “Victor Babes” University of Medicine and Pharmacy Timisoara, E. Murgu Square, No. 2, 300041 Timisoara, Romaniatoma.alina@umft.ro (A.C.B.); novacescu.dorin@umft.ro (D.N.); flavia.zara@umft.ro (F.Z.); 3 Department of Obstetrics and Gynecology, “Victor Babes” University of Medicine and Pharmacy Timisoara, E. Murgu Square, No. 2, 300041 Timisoara, Romania; 4 Faculty of Medicine and Pharmacy, University of Oradea, 410087 Oradea, Romania; manole.alexia@student.uoradea.ro; 5 Department of Surgery 1, “Victor Babes” University of Medicine and Pharmacy Timisoara, E. Murgu Square, No. 2, 300041 Timisoara, Romania; radu.dan@umft.ro; 6 Center of Hepatobiliary and Pancreatic Surgery, “Victor Babes” University of Medicine and Pharmacy Timisoara, E. Murgu Square, No. 2, 300041 Timisoara, Romania

**Keywords:** head and neck cancer, angiogenesis, VEGF, tumor microenvironment, targeted therapy, molecular mechanisms

## Abstract

Head and neck squamous cell carcinoma (HNSCC) remains one of the most aggressive and heterogeneous malignancies worldwide, characterized by high rates of locoregional recurrence, metastatic dissemination, and therapeutic resistance. Angiogenesis plays a central role in tumor progression by supporting vascular remodeling, hypoxia adaptation, invasion, immune evasion, and metastatic spread. In HNSCC, angiogenic activation is regulated through complex interactions involving hypoxia-inducible factors, vascular endothelial growth factor (VEGF) signaling, stromal remodeling, inflammatory pathways, and epigenetic mechanisms within the tumor microenvironment. Recent evidence has also highlighted the role of non-coding RNAs, particularly microRNAs, and exosome-mediated communication in modulating angiogenic and immune-related signaling pathways. Although antiangiogenic therapies, including monoclonal antibodies and tyrosine kinase inhibitors, have demonstrated biological activity in HNSCC, their clinical efficacy remains limited by tumor heterogeneity, adaptive resistance mechanisms, toxicity, and the lack of validated predictive biomarkers. Several emerging therapeutic strategies are under preclinical or early clinical investigation in HNSCC, including miRNA-based approaches, nanoparticle-assisted delivery systems, vascular normalization concepts, and combinations with immune checkpoint inhibitors; however, robust clinical evidence for most of these strategies remains limited, and their translation to routine practice requires further validation. This review provides a comprehensive overview of the molecular mechanisms regulating angiogenesis in HNSCC and critically discusses current and emerging pharmacological strategies targeting these pathways. Particular emphasis is placed on VEGF/VEGFR signaling, the integration of miRNA and exosome biology, resistance mechanisms, and translational perspectives for biomarker-guided personalized therapy. The novelty of this review lies in the systematic integration of miRNA- and exosome-mediated angiogenic regulation, therapeutic resistance pathways, and precision medicine strategies into a unified pharmacological framework, addressing gaps not fully covered by prior reviews focused primarily on VEGF-targeted agents.

## 1. Introduction

Head and neck squamous cell carcinoma (HNSCC) remains among the most aggressive and heterogeneous malignancies globally, with recent estimates indicating over 890,000 newly diagnosed cases and more than 450,000 cancer-related deaths each year [1,2]. Despite notable advancements in surgical methods, radiotherapy, and systemic therapies, the five-year survival rate for advanced-stage HNSCC has shown minimal improvement in recent decades, primarily due to high rates of local recurrence, distant metastasis, and therapeutic resistance [3,4]. HNSCC’s biological complexity is compounded by pronounced molecular heterogeneity, chronic inflammatory pathways, carcinogen exposure from tobacco and alcohol, and a rising incidence of human papillomavirus (HPV)-associated tumors, which demonstrate unique molecular and clinical profiles [5,6].

Among the hallmarks of cancer, angiogenesis is a key factor in HNSCC initiation, progression, invasion, and metastatic potential [7]. Tumor angiogenesis involves the formation of new blood vessels from pre-existing vasculature, ensuring continuous oxygen and nutrient supply to proliferating neoplastic cells [8]. In HNSCC, the angiogenic switch is primarily activated by hypoxia-induced molecular pathways, especially through hypoxia-inducible factor-1 alpha (HIF-1α) and vascular endothelial growth factor (VEGF) signaling [9,10]. Increased VEGF expression correlates with greater tumor vascularization, lymph node metastasis, poor treatment response, and unfavorable prognosis in patients with head and neck cancers [11]. Recent studies show that angiogenesis is a multifaceted process, driven by dynamic interactions among tumor cells, endothelial cells, cancer-associated fibroblasts, immune infiltrates, extracellular matrix components, and various soluble mediators within the tumor microenvironment (TME) [12].

The increasing understanding of the molecular mechanisms underlying angiogenic signaling has led to significant interest in developing antiangiogenic therapies for cancer, including HNSCC [13]. Pharmacological inhibition of VEGF and related pathways has become an important therapeutic strategy, with monoclonal antibodies against VEGF or its receptors, tyrosine kinase inhibitors, and combinational regimens integrating antiangiogenic agents with chemotherapy, radiotherapy, or immune checkpoint inhibitors showing promising results [14,15]. However, the clinical benefits of antiangiogenic therapy in HNSCC are often limited by both intrinsic and acquired resistance mechanisms, tumor heterogeneity, adaptive metabolic reprogramming, and an immunosuppressive tumor microenvironment [14,16]. It is important to distinguish agents supported by clinical trial evidence from those primarily backed by mechanistic or preclinical rationale. Bevacizumab has been the most extensively tested antiangiogenic agent in clinical trials in HNSCC but has not achieved consistent benefit or regulatory approval in this tumor type [14,17]. Sorafenib, sunitinib, and pazopanib have demonstrated limited activity as monotherapies in unselected HNSCC populations, while lenvatinib has shown more promising antiangiogenic breadth in systematic evaluations [17,18]. By contrast, strategies such as miRNA-based delivery, exosome engineering, nanoparticle platforms, and antiangiogenic–immunotherapy combinations are currently based primarily on mechanistic rationale or early preclinical findings, with only limited early-phase clinical evidence in HNSCC [17,19,20]. These challenges underscore the need to identify new molecular targets and develop more personalized treatment approaches for patients with HNSCC [21].

Recent advances in molecular oncology have highlighted the critical contribution of epigenetic regulation and non-coding RNAs, particularly microRNAs (miRNAs), in modulating angiogenic and invasive pathways in HNSCC. Dysregulated miRNAs participate in endothelial proliferation, epithelial–mesenchymal transition, tumor invasion, metastatic dissemination and resistance to systemic therapies, in part through direct and indirect regulation of VEGF- and PI3K/AKT-associated signaling cascades [22,23,24]. In addition, exosome-mediated intercellular communication has emerged as a key mechanism that supports angiogenic remodeling, immune modulation and stromal reprogramming within the HNSCC tumor microenvironment via transfer of oncogenic or tumor-suppressive miRNAs [25,26,27]. These insights have opened new perspectives for miRNA-based therapeutic strategies, including exosome- and nanoparticle-mediated delivery systems and multimodal targeted approaches, aiming to enhance treatment efficacy while limiting systemic toxicity [22,28,29].

A comprehensive literature search was conducted using the PubMed, Scopus, Web of Science, and Google Scholar databases to identify relevant studies published up to June 2025. The search strategy employed combinations of the following MeSH terms and free-text keywords: “head and neck squamous cell carcinoma”, “angiogenesis”, “VEGF”, “VEGFR”, “tumor microenvironment”, “hypoxia”, “HIF-1α”, “microRNA”, “exosomes”, “antiangiogenic therapy”, “tyrosine kinase inhibitor”, “bevacizumab”, “immunotherapy”, and “nanoparticle drug delivery”. This is a narrative review; inclusion criteria comprised peer-reviewed original research articles, systematic reviews, meta-analyses, clinical trials, and high-impact review articles published in English addressing the molecular mechanisms of tumor angiogenesis and pharmacological targeting strategies in HNSCC. Studies investigating angiogenesis in non-squamous head and neck tumors, purely non-human model systems without translational data, or languages other than English were excluded. The final literature search was completed in June 2025. Additional relevant publications were identified through manual screening of reference lists of selected articles.

Squamous cell carcinoma arises at multiple anatomical sites beyond the head and neck, including the lung, esophagus, cervix, skin, and anus. The biological rationale for focusing specifically on HNSCC—rather than SCC as a general disease class—rests on several distinctive molecular and clinical features. HNSCC is unique in its etiological heterogeneity, encompassing tumors driven by tobacco and alcohol carcinogens and a rapidly growing subset associated with oncogenic human papillomavirus (HPV) infection, each with distinct molecular profiles, immune environments, and clinical outcomes [5,6]. HPV-positive oropharyngeal SCC is characterized by chromosomal stability, immune infiltration, and favorable prognosis, while HPV-negative tumors more often display TP53 mutations, genomic instability, and greater therapeutic resistance [30]. Furthermore, the anatomical location of HNSCC creates unique therapeutic constraints, including proximity to critical neurovascular structures, radiation-induced mucosal and vascular damage, and a high incidence of trismus, dysphagia, and malnutrition that limit treatment tolerance and the feasibility of combining antiangiogenic agents with surgery or radiotherapy [17,31]. These features distinguish HNSCC from SCC at other sites and justify dedicated pharmacological research into antiangiogenic strategies specifically tailored to this anatomical and biological context.

Based on the currently available evidence, the present review aims to provide a comprehensive and critical overview of the molecular pathways regulating angiogenesis in HNSCC and the current pharmacological strategies targeting these mechanisms. Particular attention is given to VEGF-mediated signaling, hypoxia-driven molecular adaptations, tumor microenvironment interactions, epigenetic and microRNA regulation, as well as novel antiangiogenic therapeutic approaches. Furthermore, this review discusses current limitations, mechanisms of therapeutic resistance, translational challenges, and future perspectives regarding precision medicine strategies in head and neck oncology.

## 2. Biological Basis of Angiogenesis in Head and Neck Cancer

### 2.1. The Angiogenic Switch in HNSCC

The angiogenic switch is a pivotal event in the biological progression of HNSCC, reflecting the transition from a relatively growth-restricted epithelial lesion to a vascularized tumor phenotype capable of sustained expansion, local invasion, and metastatic dissemination. Under physiological conditions, angiogenesis is maintained through a tightly regulated balance between proangiogenic and antiangiogenic mediators. During carcinogenesis, this equilibrium is progressively disrupted by oncogenic activation, hypoxic stress, inflammatory signaling, extracellular matrix remodeling, and stromal–tumor interactions, leading to persistent endothelial activation and the formation of an abnormal tumor-associated vascular network [31,32].

In HNSCC, angiogenic activation may occur early in the multistep process of malignant transformation. Studies assessing microvascular density and hypoxia-related markers in premalignant and intraepithelial lesions of the upper aerodigestive tract suggest that vascular remodeling can precede overt invasion, although the extent and timing of this process may vary according to anatomical site, histological grade, and local inflammatory context [33]. In oral potentially malignant disorders and oral squamous cell carcinoma, increased expression of VEGF, FGF-2, and angiopoietin-2 has been associated not only with neovascularization, but also with epithelial–mesenchymal transition (EMT), supporting the concept that angiogenesis and invasive reprogramming are biologically interconnected processes during squamous carcinogenesis [34].

Among the central mechanisms driving the angiogenic switch in HNSCC, hypoxia has a particularly important role. Rapid proliferation of malignant cells can exceed the oxygen supply provided by the pre-existing vasculature, generating hypoxic tumor regions. Hypoxia stabilizes HIF-1α, which translocates to the nucleus and activates genes involved in angiogenesis, glycolytic metabolism, survival, invasion, and adaptation to therapeutic stress. One of the most important downstream targets of HIF-1α is VEGF, a key mediator of endothelial proliferation, migration, vascular permeability, and neovessel formation [31,35]. In addition to VEGF, hypoxia and tumor-associated inflammation may enhance the expression of other angiogenic and matrix-remodeling mediators, including platelet-derived growth factor (PDGF), fibroblast growth factors (FGFs), angiopoietins, interleukin-8 (IL-8), and matrix metalloproteinases (MMPs), thereby amplifying the vascular and invasive phenotype of HNSCC [31,36,37].

VEGF remains the most extensively investigated angiogenic marker in HNSCC. Immunohistochemical and circulating biomarker studies have shown that increased VEGF expression or elevated pretreatment VEGF levels may correlate with advanced clinical stage, poorer locoregional control, reduced event-free survival, or unfavorable overall survival, although results vary depending on tumor site, HPV status, sample type, and methodological approach [35,38,39]. The angiogenic profile of HNSCC is also heterogeneous. For example, HPV-negative tumors may show stronger associations between EGFR-related signaling and VEGF expression than HPV-positive tumors, suggesting that the molecular regulation of angiogenesis differs across biological subgroups of HNSCC [40]. This heterogeneity may partially explain why antiangiogenic therapies have shown biological promise but only limited and context-dependent clinical efficacy in unselected HNSCC populations [31,41].

The angiogenic switch in HNSCC should not be interpreted as a tumor-cell-autonomous process. Rather, it emerges from reciprocal interactions between malignant epithelial cells and the surrounding tumor microenvironment. Cancer-associated fibroblasts, tumor-associated macrophages, endothelial cells, pericytes, extracellular matrix components, and infiltrating inflammatory cells contribute to the secretion of cytokines, chemokines, and growth factors that sustain angiogenic signaling [42]. IL-8 and VEGF, for instance, may be coexpressed in HNSCC tissues and have been associated with more aggressive clinical behavior, supporting the link between inflammation, angiogenesis, and tumor progression [37]. Moreover, hypoxia can promote extracellular matrix degradation, EMT, immune evasion, and recruitment of immunosuppressive cellular populations, thereby connecting angiogenesis with invasion, metastatic competence, and resistance to antitumor immunity [33,42].

Despite the development of an apparently extensive vascular network, tumor-associated vessels in HNSCC are structurally and functionally abnormal. They are often tortuous, irregularly distributed, hyperpermeable, and incompletely covered by pericytes, resulting in heterogeneous perfusion and persistent intratumoral hypoxia. This dysfunctional vasculature can impair drug delivery, increase interstitial fluid pressure, and contribute to resistance to radiotherapy, chemotherapy, and immunotherapy [31,35,41]. Hypoxia-related biomarkers such as CA IX and GLUT-1, as well as inflammatory mediators such as IL-6, have been associated with treatment response and prognosis in patients with HNSCC treated with radiotherapy or chemoradiotherapy, further supporting the clinical relevance of the hypoxia–angiogenesis axis [35].

Therefore, the angiogenic switch represents more than a mechanism of tumor vascular supply; it is an integrated biological program linking hypoxia, inflammation, stromal remodeling, EMT, immune suppression, and therapeutic resistance. A more precise understanding of angiogenic activation in HNSCC, including its variation according to tumor site, HPV status, molecular subtype, and microenvironmental context, may help identify patients who could benefit from antiangiogenic agents, vascular-normalizing strategies, or combined approaches integrating antiangiogenic therapy with chemotherapy, radiotherapy, targeted therapy, or immune checkpoint inhibition [31,40,41].

### 2.2. VEGF Signaling Pathway

The VEGF signaling pathway represents one of the most relevant molecular systems involved in tumor-associated vascular remodeling in HNSCC. Among VEGF family members, VEGF-A is the best-characterized ligand and is generally considered the dominant mediator of angiogenic signaling in solid tumors. Its biological effects are mainly exerted through binding to VEGF receptor-1 (VEGFR-1/Flt-1) and VEGF receptor-2 (VEGFR-2/KDR/Flk-1), two receptor tyrosine kinases expressed primarily by endothelial cells, but also detectable in some tumor and stromal cell populations. In HNSCC, VEGF overexpression has been repeatedly associated with aggressive tumor behavior and adverse prognosis, although the strength of this association may vary between studies due to differences in tumor site, HPV status, treatment modality, and detection methods [31,38,43].

VEGF-A is produced by malignant epithelial cells as well as by stromal and inflammatory components of the tumor microenvironment. After secretion, VEGF-A binds to VEGFRs and induces receptor dimerization and autophosphorylation, initiating intracellular signaling cascades that regulate endothelial cell survival, proliferation, migration, vascular permeability, and new vessel formation. VEGF-A is therefore not only a marker of angiogenic activation, but also a functional driver of vascular expansion and remodeling in HNSCC [31,33]. A meta-analysis evaluating VEGF immunohistochemical expression in HNSCC reported that VEGF positivity was associated with worse 2-year overall survival, supporting its potential prognostic relevance in this tumor group [43]. However, VEGF expression alone is not sufficient to fully characterize angiogenic activity, because vascular remodeling also depends on receptor availability, downstream pathway activation, stromal interactions, and compensatory proangiogenic mechanisms [17,31].

VEGFR-1 has a more complex and context-dependent role than VEGFR-2. Although VEGFR-1 binds VEGF-A with high affinity, its kinase activity is relatively weak compared with VEGFR-2. For this reason, VEGFR-1 may function both as a modulator of VEGF bioavailability and as a signaling receptor involved in endothelial cell migration, monocyte/macrophage recruitment, and tumor–stroma communication. In the HNSCC microenvironment, this is particularly relevant because inflammatory cells can contribute to angiogenic amplification and may create a permissive niche for tumor progression [31,44]. Thus, VEGFR-1 should not be regarded merely as a passive VEGF-binding receptor, but rather as a regulatory component that links angiogenesis with inflammation and stromal remodeling.

VEGFR-2 is considered the principal mediator of the classical angiogenic effects of VEGF-A. Activation of VEGFR-2 promotes endothelial proliferation, migration, survival, vascular sprouting, and increased vascular permeability. These effects are mediated through several downstream pathways, among which PI3K/AKT and MAPK/ERK are particularly important. In HNSCC and oral squamous cell carcinoma, activation of these signaling networks contributes not only to vascular endothelial responses, but also to tumor cell survival, invasion, epithelial–mesenchymal transition, and resistance to therapy [33,45,46].

The PI3K/AKT pathway is one of the central survival pathways activated downstream of growth factor receptors, including VEGFRs and EGFR. Following receptor activation, phosphoinositide 3-kinase (PI3K) generates lipid second messengers that recruit and activate AKT. Activated AKT promotes cell survival by inhibiting pro-apoptotic mechanisms and supports cellular proliferation, metabolism, protein synthesis, and adaptation to stress. In HNSCC, dysregulation of the PI3K/AKT/mTOR axis is frequent and may result from receptor tyrosine kinase activation, PIK3CA mutations or amplification, PTEN loss, and crosstalk with other oncogenic pathways [45,46,47]. From an angiogenic perspective, PI3K/AKT signaling supports endothelial cell survival and vascular stabilization, while in tumor cells it contributes to proliferation, invasion, and resistance to radiotherapy and systemic therapy [45,46].

The MAPK/ERK pathway is another major signaling cascade activated after VEGF-A/VEGFR-2 engagement. Through the sequential activation of RAS, RAF, MEK, and ERK, this pathway regulates gene transcription programs involved in endothelial proliferation, migration, and differentiation. In the context of HNSCC, MAPK/ERK signaling is also functionally connected to EGFR activation, tumor growth, EMT, and resistance mechanisms [33,40]. The convergence of VEGF/VEGFR, EGFR, PI3K/AKT, and MAPK/ERK signaling creates a complex molecular network in which angiogenesis, tumor cell proliferation, and microenvironmental adaptation are interdependent processes rather than isolated events [33,40,45].

Importantly, the VEGF pathway does not operate as a linear and isolated signaling axis. In HNSCC, angiogenic signaling interacts with EGFR, HGF/MET, PDGF, FGF, angiopoietin, and immune-related pathways. This network-level complexity may explain why anti-VEGF or anti-VEGFR strategies have produced variable results in clinical studies. Systematic evaluations of angiogenesis inhibitors in HNSCC show that, although some agents such as bevacizumab, lenvatinib, endostatin, and multi-tyrosine kinase inhibitors have demonstrated biological or clinical activity in selected settings, their overall benefit has remained inconsistent and toxicity may limit their use [17,31]. These findings suggest that effective targeting of VEGF signaling in HNSCC may require biomarker-guided selection and rational combinations with radiotherapy, chemotherapy, EGFR inhibition, PI3K/AKT/mTOR inhibitors, or immunotherapy [17,41,46].

The major angiogenic signaling pathways activated in HNSCC, their principal molecular mediators, downstream biological effects, and current therapeutic relevance are summarized in Table 1. Other tumor-suppressive miRNAs, including miR-200c, miR-34a and miR-203, have also been implicated in EMT regulation, tumor growth control or exosome-mediated therapeutic strategies, further broadening the translational relevance of miRNA-based approaches in HNSCC.

**Figure 1:**
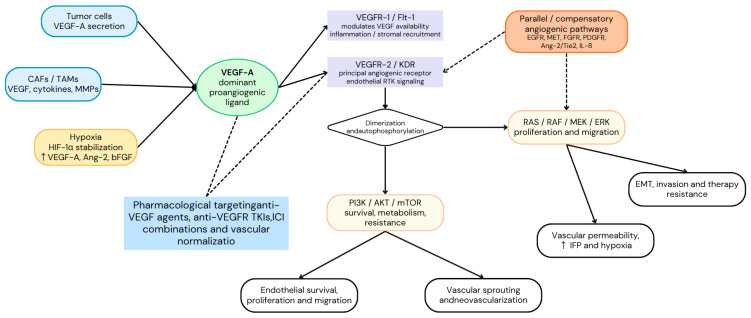
VEGF-mediated angiogenic signaling and pathway crosstalk in head and neck squamous cell carcinoma. Tumor cells, cancer-associated fibroblasts, tumor-associated macrophages, and hypoxia-driven HIF-1α stabilization contribute to the production of proangiogenic mediators, particularly VEGF-A. VEGF-A acts as the dominant proangiogenic ligand and signals mainly through VEGFR-2/KDR, the principal angiogenic receptor involved in endothelial receptor tyrosine kinase signaling. VEGFR-2 activation induces receptor dimerization and autophosphorylation, followed by downstream activation of the PI3K/AKT/mTOR and RAS/RAF/MEK/ERK pathways. These cascades promote endothelial survival, proliferation, migration, vascular sprouting, neovascularization, vascular permeability, increased interstitial fluid pressure, hypoxia, epithelial–mesenchymal transition, invasion, and therapy resistance. VEGFR-1/Flt-1 modulates VEGF availability and contributes to inflammatory and stromal recruitment. Parallel and compensatory angiogenic pathways, including EGFR, MET, FGFR, PDGFR, Ang-2/Tie2, and IL-8, interact with the VEGF/VEGFR axis and may contribute to adaptive resistance. Pharmacological targeting of this network includes anti-VEGF agents, anti-VEGFR tyrosine kinase inhibitors, immune checkpoint inhibitor-based combinations, and vascular normalization strategies. Continuous arrows indicate direct or canonical signaling relationships, whereas dashed arrows indicate indirect, modulatory, compensatory, or pharmacological interactions. Abbreviations: Ang-2, angiopoietin-2; bFGF, basic fibroblast growth factor; CAFs, cancer-associated fibroblasts; EGFR, epidermal growth factor receptor; EMT, epithelial–mesenchymal transition; FGFR, fibroblast growth factor receptor; Flt-1, fms-like tyrosine kinase-1; HIF-1α, hypoxia-inducible factor-1 alpha; HNSCC, head and neck squamous cell carcinoma; ICI, immune checkpoint inhibitor; IFP, interstitial fluid pressure; IL-8, interleukin-8; KDR, kinase insert domain receptor; MAPK/ERK, mitogen-activated protein kinase/extracellular signal-regulated kinase pathway; MEK, mitogen-activated protein kinase kinase; MET, mesenchymal–epithelial transition factor receptor; MMPs, matrix metalloproteinases; mTOR, mechanistic target of rapamycin; PDGFR, platelet-derived growth factor receptor; PI3K/AKT, phosphoinositide 3-kinase/protein kinase B pathway; RAF, rapidly accelerated fibrosarcoma kinase; RAS, rat sarcoma virus oncogene homolog; RTK, receptor tyrosine kinase; TAMs, tumor-associated macrophages; Tie2, tyrosine kinase with immunoglobulin-like and EGF-like domains 2; TKIs, tyrosine kinase inhibitors; VEGF-A, vascular endothelial growth factor A; VEGFR, vascular endothelial growth factor receptor.

**Figure 2:**
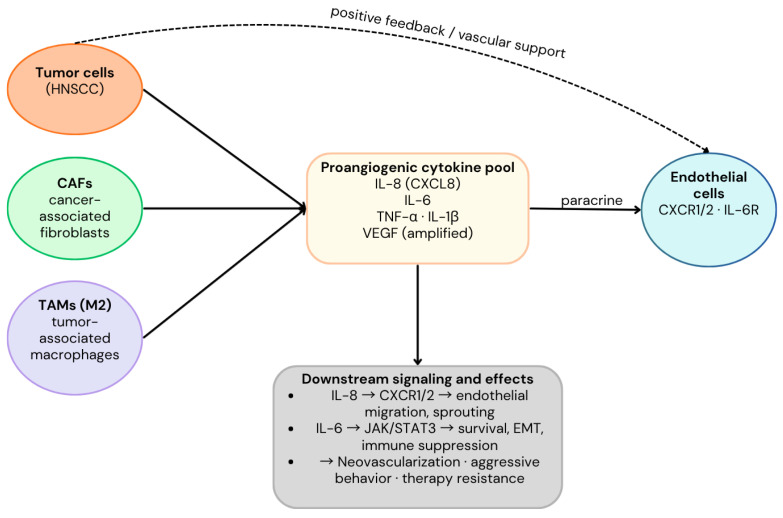
Inflammatory cytokine axis in the head and neck squamous cell carcinoma (HNSCC) microenvironment. Tumor cells, cancer-associated fibroblasts (CAFs), and tumor-associated macrophages (TAMs) secrete proinflammatory and proangiogenic cytokines, including interleukin-8 (IL-8/CXCL8), interleukin-6 (IL-6), tumor necrosis factor-alpha (TNF-α), and interleukin-1 beta (IL-1β), which together amplify vascular endothelial growth factor (VEGF) signaling. IL-8 acts on endothelial CXCR1/2 receptors to promote endothelial migration and vascular sprouting, whereas IL-6 activates JAK/STAT3 signaling to support survival, epithelial–mesenchymal transition (EMT), and immune suppression. These interactions sustain neovascularization, aggressive tumor behavior, and therapy resistance, partly independently of the classical VEGF/VEGFR pathway. Solid arrows indicate direct secretion or canonical signaling; the dashed arrow indicates indirect or feedback interactions. Abbreviations: CAFs, cancer-associated fibroblasts; CXCR1/2, C-X-C chemokine receptor types 1 and 2; EMT, epithelial–mesenchymal transition; IL, interleukin; JAK, Janus kinase; STAT3, signal transducer and activator of transcription 3; TAMs, tumor-associated macrophages; TNF-α, tumor necrosis factor-alpha; VEGF, vascular endothelial growth factor. Legend: solid arrows- direct secretion/canonical signaling; dashed arrow indirect/feedback interactions.

While Table 1 provides a comparative overview of the major angiogenic pathways involved in HNSCC, Figure 1 focuses specifically on the central VEGF-A/VEGFR signaling axis, highlighting its cellular sources, receptor-mediated activation, downstream cascades, and biological consequences. To aid readers less familiar with the inflammatory and stromal angiogenic axes, a supplementary schematic (Figure 2) illustrating the inflammatory cytokine axis—particularly the IL-8/CXCR2, IL-6/STAT3, and TAM-mediated proangiogenic signaling network in the HNSCC microenvironment—has been added. This figure specifically addresses the crosstalk between tumor-associated macrophages, cancer-associated fibroblasts, and endothelial cells in sustaining angiogenesis independently of the classical VEGF pathway, providing visual context for items in Table 1 not covered by Figure 1.

The VEGF-A/VEGFR axis is a major regulator of angiogenic signaling in HNSCC. VEGFR-2 primarily mediates endothelial proliferation, migration, permeability, and sprouting, whereas VEGFR-1 appears to modulate VEGF availability and contribute to inflammatory and stromal components of the angiogenic response. Downstream activation of PI3K/AKT and MAPK/ERK links VEGF signaling to endothelial survival, tumor progression, EMT, and treatment resistance. Therefore, this pathway remains biologically relevant not only as a mechanism of tumor vascularization, but also as a potential therapeutic target within multimodal and molecularly stratified treatment strategies.

### 2.3. Hypoxia-Inducible Factors and Tumor Progression

Hypoxia-inducible factors are central regulators of tumor adaptation to oxygen deprivation and have particular relevance in HNSCC, where hypoxic regions are frequently associated with aggressive biological behavior and reduced sensitivity to conventional therapies. Among these transcription factors, HIF-1α is the most extensively studied. Under normoxic conditions, HIF-1α is hydroxylated by prolyl hydroxylase domain enzymes and rapidly degraded through the von Hippel–Lindau/proteasomal pathway. In hypoxic conditions, this degradation is inhibited, allowing HIF-1α stabilization, nuclear translocation, dimerization with HIF-1β, and activation of genes involved in metabolic adaptation, angiogenic remodeling, invasion, and cellular survival [48,49].

A major consequence of HIF-1α activation is metabolic reprogramming. Hypoxic tumor cells shift toward glycolysis and increase the expression of glucose transporters and glycolytic enzymes, enabling ATP production even when oxygen availability is limited. This metabolic plasticity supports tumor cell survival in poorly perfused regions and contributes to acidosis, extracellular matrix remodeling, and invasive behavior [49,50]. In HNSCC, hypoxia-related markers such as GLUT-1 and CA IX have been investigated as indicators of hypoxic adaptation and have been associated with treatment response and prognosis, particularly in patients receiving radiotherapy or chemoradiotherapy [35].

HIF-1α also contributes to tumor progression by coordinating proangiogenic and invasive programs. Beyond stimulating angiogenic mediators, HIF signaling promotes phenotypic changes linked to epithelial–mesenchymal transition, migration, extracellular matrix degradation, and metastatic competence. In oral squamous cell carcinoma, increased HIF-1α expression has been reported during the transition from dysplastic lesions to invasive carcinoma and has been associated with larger tumor size, lymph node metastasis, advanced clinical stage, and poorer survival in some cohorts [51]. However, the prognostic value of HIF-1α is not completely uniform across all head and neck subsites. Large cohort analyses suggest that its clinical impact may differ between oral cavity, oropharyngeal, and laryngeal squamous cell carcinomas, emphasizing the importance of tumor site and biological context when interpreting HIF-1α expression [52].

From a therapeutic perspective, hypoxia-driven HIF activation is clinically important because it can reduce radiosensitivity, support resistance to systemic therapy, and favor selection of more aggressive tumor cell populations. These effects are mediated not only through angiogenic and metabolic pathways, but also through interactions with DNA repair mechanisms, stemness-associated programs, immune suppression, and adaptive stress responses [49,53]. Therefore, HIF-1α should be considered not only a marker of hypoxia, but also a functional mediator of tumor progression and treatment resistance in HNSCC. Its integration with metabolic, angiogenic, and immune biomarkers may improve future patient stratification and help guide combined therapeutic strategies targeting the hypoxic tumor microenvironment.

### 2.4. Crosstalk Between Tumor Cells and the Microenvironment

The progression of HNSCC is strongly influenced by reciprocal communication between malignant epithelial cells and the surrounding tumor microenvironment. This microenvironment includes cancer-associated fibroblasts (CAFs), tumor-associated macrophages (TAMs), endothelial cells, inflammatory infiltrates, extracellular matrix components, soluble mediators, and extracellular vesicles. Rather than acting as a passive stromal compartment, the tumor microenvironment actively regulates angiogenesis, invasion, immune escape, metabolic adaptation, and therapeutic response [54].

CAFs represent one of the most abundant stromal populations in HNSCC and contribute to tumor progression through extracellular matrix remodeling, secretion of growth factors, cytokines, chemokines, and modulation of immune cell recruitment. Their density has been associated with advanced T stage, nodal involvement, vascular and perineural invasion, increased proliferative activity, and higher risk of local recurrence, supporting their role as clinically relevant stromal markers [55]. Functionally, CAFs can reinforce proangiogenic signaling by producing mediators that support endothelial activation and by creating a remodeled matrix permissive for vascular sprouting and tumor cell migration [56].

TAMs are another essential component of the HNSCC microenvironment. Although macrophages may display heterogeneous phenotypes, enrichment of total TAMs and particularly M2-like macrophages has been associated with advanced tumor stage, nodal positivity, vascular invasion, lymphatic invasion, and poorer survival outcomes [57,58]. These cells support tumor progression through secretion of immunosuppressive and proangiogenic mediators, including IL-10, TGF-β, VEGF, IL-6, and other inflammatory cytokines. Through these mechanisms, TAMs contribute to immune evasion, extracellular matrix remodeling, angiogenesis, epithelial–mesenchymal transition, and resistance to treatment [58,59].

Inflammatory cells within the HNSCC microenvironment further amplify tumor-supportive signaling. T lymphocytes, neutrophils, dendritic cells, myeloid-derived suppressor cells, and regulatory T cells form a dynamic immune network that may either restrain or promote tumor growth depending on their functional state. In many advanced tumors, chronic inflammation favors a suppressive immune contexture characterized by impaired cytotoxic activity, accumulation of regulatory populations, and secretion of cytokines that sustain angiogenesis and stromal activation [54,60]. This inflammatory milieu helps explain why angiogenesis, immune evasion, and treatment resistance are closely interconnected in HNSCC.

Exosomes represent an additional layer of intercellular communication in the HNSCC microenvironment, enabling tumor cells, CAFs, and immune cells to exchange microRNAs, proteins, and other bioactive cargo. Through this vesicular transfer, exosomal signaling modifies endothelial behavior, macrophage polarization, fibroblast activation, and immune-cell function, thereby connecting angiogenic remodeling with immune escape and stromal reprogramming. The molecular mechanisms and therapeutic implications of exosome-mediated angiogenic regulation are discussed in detail in Section 3.3 [61,62].

The crosstalk between tumor cells and the microenvironment thus constitutes a self-reinforcing network in which CAFs, TAMs, inflammatory cells, and extracellular vesicles cooperate to sustain angiogenesis, invasion, and treatment resistance. This multicellular circuit provides the biological foundation for the molecular regulatory mechanisms described in Section 3 and the pharmacological targeting strategies discussed in Section 4.

## 3. Molecular Regulation of Tumor Angiogenesis

Tumor angiogenesis in HNSCC is regulated by a complex molecular network that integrates genetic alterations, epigenetic remodeling, transcriptional control, non-coding RNAs, and microenvironment-derived signals. These mechanisms do not act independently, but converge to regulate endothelial activation, stromal remodeling, immune escape, metabolic adaptation, and resistance to anticancer therapies. Understanding the molecular regulation of angiogenesis is therefore essential for identifying predictive biomarkers and for developing more precise antiangiogenic and combination therapeutic strategies.

### 3.1. Genetic and Epigenetic Regulation

Genetic alterations contribute to angiogenic regulation in HNSCC by modifying signaling pathways involved in tumor growth, stromal interaction, hypoxic adaptation, and vascular remodeling. Although angiogenesis is rarely driven by a single mutation, variants affecting angiogenesis-related genes may influence disease progression and treatment outcome. Germline polymorphisms in VEGF/VEGFR, ANGPT/TEK, FGF/FGFR, PDGF/PDGFR, MMP, and TIMP-related pathways have been associated with differences in locoregional control, metastasis risk, survival, or response to radiotherapy and chemoradiotherapy in HNSCC cohorts [36,63]. These findings suggest that inherited genetic variability may modulate the angiogenic phenotype and partially explain interpatient heterogeneity in therapeutic response.

Somatic alterations in oncogenic and tumor suppressor pathways also influence angiogenesis indirectly. Mutations in genes such as TP53, PIK3CA, NOTCH1, and alterations in EGFR-associated signaling can modify transcriptional programs related to proliferation, inflammation, hypoxia tolerance, and growth factor production. For example, TP53 dysfunction may favor a proangiogenic state by reducing the transcriptional repression of angiogenic mediators and by promoting genomic instability and inflammatory signaling. Similarly, activation of PI3K-associated pathways can increase the expression of genes involved in survival, metabolism, and vascular remodeling, thereby connecting oncogenic transformation with angiogenic competence [64,65].

Transcriptional regulation represents another key level of angiogenic control. In HNSCC, transcription factors activated by hypoxia, inflammation, oxidative stress, and oncogenic receptor signaling coordinate the expression of angiogenesis-related genes. These transcriptional networks regulate not only VEGF-family members, but also angiopoietins, chemokines, matrix-remodeling enzymes, and immune-modulatory mediators. Importantly, transcriptional angiogenic programs differ according to HPV status, tumor site, stromal composition, and immune infiltration, emphasizing that angiogenesis in HNSCC should be interpreted as a context-dependent molecular phenotype rather than a uniform process [30,66].

Epigenetic regulation further refines angiogenic signaling by altering gene expression without changing the DNA sequence. DNA methylation, histone modifications, chromatin remodeling, and RNA methylation can silence tumor suppressor genes, activate oncogenic transcriptional programs, and reshape the interaction between tumor cells and the microenvironment [67]. Aberrant promoter hypermethylation may suppress genes involved in cell-cycle control, apoptosis, immune regulation, and vascular homeostasis, whereas global hypomethylation may contribute to genomic instability and transcriptional plasticity. In HNSCC, methylation patterns differ according to HPV status and clinicopathological features, supporting their potential use as biomarkers for molecular classification and prognosis [66,68].

DNA methyltransferase-related alterations may also contribute to HNSCC biology. Analyses of DNMT-family genes have identified amplifications, deletions, and mutations that may affect epigenetic stability and gene-expression control [69]. In addition to DNA methylation, RNA epigenetic modifications such as m6A and m5C have emerged as regulators of HNSCC progression, with several methylation-related regulators showing prognostic value [70,71]. Although their direct role in angiogenesis requires further validation, these modifications may influence angiogenic pathways indirectly by regulating mRNA stability, translation, immune signaling, and stress-adaptation programs.

The genetic and epigenetic regulation of tumor angiogenesis in HNSCC involves a multilayered system in which inherited variants, somatic mutations, transcriptional programs, DNA methylation, and RNA modifications interact to shape the angiogenic phenotype. These mechanisms may help explain why angiogenic activity and response to antiangiogenic therapies vary markedly between patients. From a pharmacological perspective, these epigenetic mechanisms have direct therapeutic relevance: aberrant DNA methylation and histone modifications can silence antiangiogenic regulators or activate proangiogenic gene programs, thereby reducing the efficacy of VEGF-targeted agents and contributing to adaptive resistance. Epigenetic therapies such as DNA methyltransferase inhibitors and histone deacetylase (HDAC) inhibitors have been proposed as combination partners for antiangiogenic strategies, with the rationale that restoring epigenetic control may sensitize tumor vasculature and immune infiltrates to pharmacological targeting [66,67,68]. Although direct evidence for these combinations in HNSCC remains limited, integrating epigenomic profiling with angiogenic biomarkers offers a promising direction for precision treatment stratification. Future translational studies should therefore integrate genomic and epigenomic profiling with angiogenic and immune biomarkers to improve patient stratification and guide personalized treatment strategies.

### 3.2. microRNAs Involved in Angiogenesis

MicroRNAs are important post-transcriptional regulators of angiogenic signaling in HNSCC, acting either as oncogenic miRNAs or tumor-suppressive miRNAs depending on their molecular targets and cellular context. By binding to complementary sequences in target mRNAs, they can regulate endothelial proliferation, tumor cell invasion, epithelial–mesenchymal transition, immune modulation, and response to chemo- or radiotherapy. In HNSCC, altered miRNA profiles have been identified in tumor tissue, saliva, plasma, serum, and exosomes, supporting their potential value as minimally invasive biomarkers and as candidates for molecularly guided therapies [72,73].

Among the most consistently reported oncogenic miRNAs, miR-21 has a central role in HNSCC progression. It is frequently overexpressed and has been linked to tumor proliferation, invasion, EMT, angiogenic signaling, and resistance to therapy. Mechanistically, miR-21 may promote malignant behavior by suppressing tumor-suppressive targets involved in apoptosis, cell-cycle control, and PI3K/AKT-related signaling. In the tumor microenvironment, miR-21 may also be transferred through exosomes, including CAF-derived vesicles, thereby reinforcing stromal activation, invasion, and resistance mechanisms [62,74]. Its recurrent association with poor prognosis and treatment response has made miR-21 one of the most investigated miRNAs in HNSCC biomarker studies [75].

miR-155 is another relevant miRNA in head and neck carcinogenesis, particularly because of its relationship with inflammation, immune regulation, and tumor progression. miR-155 has been reported among dysregulated miRNAs in HNSCC and may contribute to oncogenic signaling by modulating pathways involved in cell survival, immune escape, and inflammatory activation [75,76]. Although its direct role in endothelial proliferation is less clearly established than that of VEGF-associated mediators, miR-155 may indirectly support a proangiogenic phenotype by shaping inflammatory cytokine networks and immune-cell behavior within the tumor microenvironment.

miR-146a has a more context-dependent function. It is closely linked to inflammatory and NF-κB-associated signaling and may act either as a negative regulator of excessive inflammation or as a contributor to tumor adaptation depending on tumor type and biological context. In HNSCC, miR-146a has been discussed as part of broader non-coding RNA networks involved in tumor progression, immune regulation, and therapeutic response [73,76]. Its relevance to angiogenesis is probably indirect, mediated through inflammatory signaling, cytokine modulation, and interactions with stromal or immune cells rather than through a single linear proangiogenic pathway.

miR-221, often considered together with miR-222, has been associated with cell-cycle progression, EMT, invasion, and resistance to therapy in several malignancies. In the context of HNSCC, miR-221/222-related signaling may contribute to tumor aggressiveness by regulating tumor-suppressive targets and pathways involved in proliferation, migration, and survival. These miRNAs have also been implicated in EMT-associated drug resistance, supporting their potential relevance in tumors with invasive and treatment-resistant phenotypes [77]. Although evidence specifically linking miR-221 to angiogenesis in HNSCC remains less extensive than for miR-21, its involvement in EMT, cellular plasticity, and therapy resistance makes it relevant within the broader angiogenic and invasive regulatory network.

In addition to their individual regulatory effects, several miRNAs involved in HNSCC appear to converge on common biological processes such as EMT, inflammatory signaling, exosome-mediated intercellular communication, therapy resistance, and angiogenic remodeling. Therefore, the most relevant miRNAs discussed in this section are summarized in Table 2, together with their main biological roles, angiogenesis-related mechanisms, and potential translational relevance.

Overall, miR-21, miR-155, miR-146a, and miR-221 represent biologically relevant components of the post-transcriptional regulation of angiogenesis-associated tumor behavior in HNSCC. Their effects extend beyond endothelial proliferation alone and include modulation of invasion, EMT, cancer stemness, stromal communication, immune escape, and therapeutic resistance. The translational limitations of miRNA-based biomarkers and the requirements for their clinical validation are discussed in the context of precision oncology in Section 6.1.

### 3.3. Exosomal Communication and Angiogenic Signaling

Exosomes are small extracellular vesicles that mediate intercellular communication by transferring biologically active cargo, including microRNAs, long non-coding RNAs, circular RNAs, proteins, lipids, and DNA fragments. In HNSCC, tumor-derived exosomes contribute to the formation of a permissive microenvironment by modifying stromal cells, endothelial cells, immune cells, and distant premetastatic niches. Through this cargo transfer, exosomes can coordinate angiogenic signaling, invasion, immune suppression, metabolic adaptation, and resistance to therapy [74,78].

Tumor-derived exosomes may promote angiogenic remodeling by delivering regulatory RNAs and proteins to endothelial cells and stromal components. Exosomal cargo can enhance endothelial cell migration, tube formation, vascular permeability, and survival, thereby supporting neovascularization. In laryngeal squamous cell carcinoma, exosome-derived molecules have been linked to mechanisms involving VEGFR2-associated angiogenic signaling, glycolytic reprogramming, invasion, and xenograft growth, although the available evidence remains preliminary and methodologically heterogeneous [79]. These findings suggest that exosomal communication may function as an additional layer of angiogenic regulation beyond soluble growth factors alone.

The transport of miRNAs through exosomes is particularly relevant because the vesicular membrane protects miRNAs from enzymatic degradation, increasing their stability in biological fluids such as saliva, plasma, and serum. This makes exosomal miRNAs attractive candidates for liquid biopsy approaches in HNSCC. Salivary exosomal miRNAs have been investigated for early detection of oral cavity and oropharyngeal cancers, while serum-derived exosomal miRNAs have shown potential diagnostic and prognostic value in laryngeal carcinoma [79,80]. However, clinical implementation is still limited by small cohorts, lack of standardized isolation methods, heterogeneous detection platforms, and insufficient external validation.

Exosomal communication also links tumor cells with cancer-associated fibroblasts and immune cells. CAF-derived exosomes may transfer miRNAs that promote tumor cell invasion, EMT, metastatic potential, and therapy resistance [58]. In addition, both tumor-derived and CAF-derived exosomes can contribute to immune escape by impairing cytotoxic T-cell activity, promoting regulatory T-cell accumulation, favoring immunosuppressive macrophage phenotypes, and modulating PD-1/PD-L1-related pathways [61]. These mechanisms are important because they connect angiogenic remodeling with immune suppression and may partly explain resistance to immune checkpoint inhibitors in selected HNSCC patients.

From a therapeutic perspective, exosomes have a dual relevance. On one hand, tumor-derived exosomes may represent therapeutic targets, because blocking their release, uptake, or cargo function could disrupt tumor–stroma communication. On the other hand, engineered exosomes may serve as natural nanocarriers for delivering tumor-suppressive miRNAs or anticancer agents. For example, exosome-mediated delivery of miR-200c has been proposed as a potential strategy to inhibit EMT and improve therapeutic efficacy in HNSCC [29]. Despite this promise, several barriers remain, including exosome heterogeneity, production scalability, cargo loading efficiency, biodistribution, safety, and reproducibility.

Exosomal communication represents an important regulatory mechanism in HNSCC angiogenesis and tumor progression. By transporting miRNAs and other molecular cargos, exosomes integrate angiogenic signaling with EMT, immune escape, stromal activation, and treatment resistance. Their future clinical value will depend on standardized extracellular vesicle workflows, validated biomarker panels, and carefully designed translational studies assessing both diagnostic and therapeutic applications.

## 4. Pharmacological Targeting of Angiogenesis

Pharmacological targeting of angiogenesis in HNSCC is based on the rationale that tumor vascularization supports growth, invasion, metastatic dissemination, and resistance to therapy. Antiangiogenic agents have therefore been investigated as monotherapy or, more frequently, in combination with chemotherapy, radiotherapy, EGFR inhibitors, or immunotherapy. However, unlike in several other solid tumors, the clinical translation of antiangiogenic therapy in HNSCC has remained limited, mainly because of tumor heterogeneity, compensatory angiogenic pathways, toxicity, and the lack of validated predictive biomarkers [17]. To provide a systematic overview of the clinical evidence supporting antiangiogenic strategies in HNSCC, the main completed and ongoing clinical trials are summarized in Table 3, including agent, target, clinical phase, patient population, treatment combination, key efficacy outcomes, principal toxicities, and overall conclusion. This tabular overview complements the narrative discussion in Section 4.1, Section 4.2 and Section 4.3 and facilitates direct comparison of the available evidence across therapeutic classes.

### 4.1. Anti-VEGF Therapies

Anti-VEGF therapies interfere with the VEGF/VEGFR axis by preventing VEGF ligands from activating endothelial receptors or by directly blocking VEGF receptor signaling. This inhibition reduces endothelial cell proliferation, migration, vascular permeability, and neovessel formation. In theory, anti-VEGF treatment may also transiently “normalize” abnormal tumor vessels, improving oxygenation and drug delivery; however, this effect is context-dependent and difficult to predict clinically [16,83].

Bevacizumab is a humanized monoclonal antibody that binds circulating VEGF-A, preventing its interaction with VEGFR-1 and VEGFR-2 on endothelial cells. It is the most extensively studied antiangiogenic agent in HNSCC. Clinical trials have evaluated bevacizumab in recurrent/metastatic disease and in combination with chemotherapy, radiotherapy, chemoradiotherapy, EGFR inhibition, or immune checkpoint blockade. A systematic review of angiogenesis inhibitors in HNSCC identified bevacizumab as the most frequently investigated agent, included in 13 trials, but concluded that most antiangiogenic studies did not demonstrate consistent clinical benefit and that bevacizumab-containing regimens were often associated with substantial toxicity [39]. Therefore, although bevacizumab has biological plausibility and encouraging results in selected combinations, it has not become a standard antiangiogenic therapy for unselected HNSCC patients.

Aflibercept is a recombinant fusion protein that acts as a soluble decoy receptor for VEGF-A, VEGF-B, and placental growth factor. By trapping multiple VEGF-family ligands, aflibercept has a broader ligand-binding profile than bevacizumab. This mechanism may theoretically reduce escape through alternative VEGF ligands, but clinical evidence supporting aflibercept in HNSCC remains sparse compared with other malignancies. Most available clinical experience with aflibercept comes from colorectal cancer and ocular vascular diseases, and its role in HNSCC remains investigational rather than established [84,85].

Ramucirumab is a fully human monoclonal antibody directed against VEGFR-2, thereby blocking receptor activation by VEGF ligands. Unlike bevacizumab and aflibercept, which target VEGF ligands, ramucirumab acts at the receptor level. It has demonstrated clinical benefit in selected solid tumors, including gastric/gastroesophageal and lung cancers, but direct evidence in HNSCC is limited. Its inclusion in discussions of HNSCC angiogenesis is mainly justified by its mechanism and by the broader interest in VEGFR-2 blockade as an antiangiogenic strategy. At present, ramucirumab should be considered experimental in HNSCC unless supported by clinical trial enrollment or future biomarker-guided evidence [86,87].

The main limitations of anti-VEGF therapy in HNSCC are biological redundancy and clinical toxicity. Tumors can bypass VEGF blockade by activating alternative angiogenic pathways (FGF, PDGF, angiopoietin/Tie2, MET, inflammatory cytokines), while antiangiogenic treatment may promote hypoxia-driven adaptation, invasion, and resistance in some contexts [16,17,88]. Clinically, relevant adverse events include hypertension, bleeding, thromboembolic events, wound-healing complications, fistula formation, proteinuria, and mucosal toxicity—particularly significant in head and neck cancer due to local tissue invasion and radiotherapy- or surgery-induced vascular damage [17,83].

Anti-VEGF therapy remains a biologically attractive but clinically challenging approach in HNSCC. Bevacizumab has the most direct clinical evidence, but its benefit is inconsistent and toxicity remains a major concern. Aflibercept and ramucirumab have clear mechanistic relevance, yet their roles in HNSCC are not established. Future progress will likely depend on biomarker-guided patient selection, rational combination regimens, and careful integration with immunotherapy, chemotherapy, radiotherapy, or molecularly targeted agents.

### 4.2. Tyrosine Kinase Inhibitors

Tyrosine kinase inhibitors (TKIs) represent an important class of antiangiogenic agents because they block intracellular signaling downstream of multiple receptor tyrosine kinases involved in tumor vascularization. Unlike monoclonal antibodies directed against VEGF or VEGFR, most antiangiogenic TKIs are orally administered small molecules with multi-target activity, commonly inhibiting VEGFRs, PDGFRs, FGFRs, c-KIT, RET, RAF, or other kinases. This broader activity may theoretically overcome single-pathway escape mechanisms, but it also increases the risk of off-target toxicity and complicates biomarker selection [17,89].

Sunitinib is a multi-target TKI that inhibits VEGFR, PDGFR, c-KIT, FLT3, and related kinases. Its antiangiogenic effect is mainly mediated through VEGFR and PDGFR blockade, leading to reduced endothelial proliferation and impaired pericyte-supported vascular maturation. Although sunitinib has demonstrated activity in several solid tumors, its efficacy in unselected HNSCC populations has been limited. In a phase II trial in recurrent/metastatic HNSCC, sunitinib at 37.5 mg/day demonstrated a partial response rate of approximately 2–13% and median progression-free survival ranging from 2 to 4 months, with significant toxicity including oral mucositis and bleeding [17,18]. These results did not support further single-agent development, and its future role is more likely to depend on combination strategies or biomarker-defined subgroups [17,18].

Sorafenib inhibits RAF kinases as well as VEGFR and PDGFR signaling, thereby combining antiangiogenic and antiproliferative effects. It has been evaluated in several malignancies and is a reference antiangiogenic TKI in hepatocellular carcinoma. However, in HNSCC, phase II studies in recurrent/metastatic disease reported objective response rates below 5% and median progression-free survival of approximately 2–4 months as monotherapy, and sorafenib has not shown sufficient activity to become standard therapy. Its use is limited by moderate efficacy, adverse events, and the availability of more effective systemic options such as immune checkpoint inhibitors in recurrent/metastatic disease [17,81].

Lenvatinib targets VEGFR1–3, FGFR1–4, PDGFRα, RET, and KIT. Its additional inhibition of FGFR signaling is particularly relevant because FGF pathways may participate in resistance to VEGF-directed therapy. Among the TKIs investigated in HNSCC, lenvatinib has attracted interest because of its broad antiangiogenic profile and potential immunomodulatory effects. In a systematic analysis of antiangiogenic agents in HNSCC, lenvatinib demonstrated a response rate of approximately 13–15% and a disease control rate exceeding 50% in early-phase studies, with a more acceptable tolerability profile compared to earlier agents [17]. Systematic analyses of angiogenesis inhibitors in HNSCC suggest that lenvatinib appears relatively promising and better tolerated than some earlier antiangiogenic approaches, although its optimal place in therapy remains investigational and requires further validation in prospective trials [17,18].

Pazopanib is another oral multi-target TKI directed mainly against VEGFR, PDGFR, and c-KIT. It has demonstrated clinical utility in renal cell carcinoma and soft tissue sarcoma, but evidence in HNSCC is limited. Its biological rationale is based on vascular inhibition and stromal modulation, yet, as with other TKIs, clinical benefit in HNSCC is expected to be highly dependent on patient selection, tolerability, and combination strategy rather than broad single-agent use [18,82].

The adverse-event profile of antiangiogenic TKIs is clinically important. Common toxicities include hypertension, fatigue, diarrhea, nausea, anorexia, weight loss, hand–foot skin reaction, mucositis, hypothyroidism, hepatotoxicity, proteinuria, bleeding, and thromboembolic complications [89,90]. Cardiovascular toxicity is particularly relevant for VEGFR-directed TKIs, with reported events including hypertension, atrial fibrillation, reduced cardiac function, heart failure, and thromboembolic events [89,91]. In HNSCC, these toxicities must be interpreted carefully because many patients have prior radiotherapy, mucosal injury, malnutrition, vascular comorbidities, or surgically altered tissues.

Resistance to TKIs may occur through multiple mechanisms, including activation of compensatory angiogenic pathways, secondary pathway reactivation, tumor microenvironment remodeling, altered drug transport, epithelial–mesenchymal transition, and selection of resistant cellular subclones [92]. In the antiangiogenic context, escape through FGF, PDGF, MET, angiopoietin/Tie2, inflammatory cytokines, and hypoxia-driven adaptation may reduce the durability of VEGFR blockade. These mechanisms support the rationale for combination therapies, particularly with immunotherapy, EGFR inhibition, radiotherapy, or agents targeting parallel angiogenic pathways [18,92].

Predictive biomarkers for TKI response in HNSCC remain insufficiently validated. Candidate markers include baseline VEGF/VEGFR expression, angiogenic cytokine signatures, FGFR pathway activation, immune infiltration patterns, hypoxia-related markers, circulating angiogenic factors, and pharmacokinetic exposure levels. However, none has yet achieved routine clinical implementation for selecting HNSCC patients for antiangiogenic TKI therapy [18,93]. Therefore, future trials should incorporate prospective biomarker testing, molecular stratification, and longitudinal monitoring of resistance to identify patients most likely to benefit from this therapeutic class.

Overall, sunitinib, sorafenib, lenvatinib, and pazopanib illustrate both the potential and the limitations of multi-target antiangiogenic therapy. Their broad kinase inhibition may be biologically useful in tumors with redundant angiogenic signaling, but clinical benefit in HNSCC remains limited without appropriate patient selection. At present, TKIs should be considered investigational or combination-based strategies in HNSCC rather than established standard antiangiogenic monotherapies.

### 4.3. Immunotherapy and Angiogenesis Interactions

The interaction between angiogenesis and antitumor immunity is increasingly recognized as a central therapeutic concept in HNSCC. Although immune checkpoint inhibitors targeting the PD-1/PD-L1 axis have changed the treatment landscape of recurrent and metastatic HNSCC, only a subset of patients achieve durable responses. Resistance to immunotherapy is partly related to the structure and function of the tumor microenvironment, including abnormal vasculature, hypoxia, reduced lymphocyte infiltration, immunosuppressive macrophages, regulatory T cells, and proangiogenic cytokine networks [19,94].

The PD-1/PD-L1 pathway allows tumor cells and immune-regulatory cells to suppress cytotoxic T-cell activity. In HNSCC, pembrolizumab and nivolumab have demonstrated clinical benefit in recurrent/metastatic disease, and PD-L1 expression, usually assessed by combined positive score, is used to guide treatment selection in several clinical settings. However, PD-L1 expression alone is an imperfect biomarker, because treatment response is influenced by additional factors such as tumor mutational burden, HPV status, immune infiltration, hypoxia, stromal composition, and other immune checkpoints [94,95].

Angiogenesis contributes to immune escape through several mechanisms. VEGF and related proangiogenic mediators can impair dendritic cell maturation, reduce T-cell trafficking into tumor tissue, promote regulatory T-cell and myeloid-derived suppressor cell accumulation, and favor tumor-associated macrophage polarization toward immunosuppressive phenotypes [96,97]. In addition, structurally abnormal tumor vessels limit effective lymphocyte extravasation and create hypoxic regions that further reinforce immune suppression. Therefore, angiogenesis is not only a vascular process, but also an immune-regulatory mechanism.

The concept of vascular normalization provides a rationale for combining antiangiogenic therapy with immunotherapy. Rather than simply eliminating blood vessels, appropriately dosed antiangiogenic treatment may transiently improve vessel structure and function, reduce hypoxia, decrease interstitial pressure, and facilitate immune-cell infiltration. This normalized vascular window may enhance the delivery and activity of immune checkpoint inhibitors, while PD-1/PD-L1 blockade may restore T-cell cytotoxicity within a more permissive tumor microenvironment [96,98].

The potential synergy between antiangiogenic therapy and immunotherapy has been demonstrated most clearly in other solid tumors, such as renal cell carcinoma and hepatocellular carcinoma, where combinations of VEGF/VEGFR-targeted agents and immune checkpoint inhibitors have become clinically important. In HNSCC, the biological rationale is strong, but the clinical evidence remains less mature. Early studies and translational data suggest that combined antiangiogenic and immune checkpoint blockade may be useful in selected patients, particularly when angiogenesis, hypoxia, and immune suppression coexist within the tumor microenvironment [19,20,98].

Despite this promise, several challenges remain. The optimal antiangiogenic agent, dose, sequence, and timing relative to immunotherapy are not yet clearly defined in HNSCC. Excessive vascular inhibition may worsen hypoxia and impair immune-cell delivery, whereas insufficient inhibition may fail to remodel the vasculature. Furthermore, combined treatment can increase toxicity, including hypertension, bleeding, thromboembolic events, immune-related adverse events, mucosal toxicity, and impaired wound healing. These risks are especially relevant in head and neck cancer patients with prior surgery, radiation-induced tissue damage, or locally advanced mucosal disease [19,98].

The interaction between immunotherapy and angiogenesis represents a promising but complex therapeutic field in HNSCC. Antiangiogenic therapy may enhance immunotherapy by promoting vascular normalization and reducing VEGF-mediated immunosuppression, while PD-1/PD-L1 blockade may restore antitumor immune activity within a remodeled tumor microenvironment. It is important to distinguish evidence derived from HNSCC-specific studies from evidence extrapolated from other tumor types. The most robust clinical data for combined antiangiogenic–immunotherapy strategies come from renal cell carcinoma and hepatocellular carcinoma, where combinations such as axitinib plus pembrolizumab and atezolizumab plus bevacizumab have demonstrated significant improvements in overall survival [96,98]. In HNSCC, the published evidence remains less mature: early-phase and retrospective data suggest potential activity of anti-PD-1 agents combined with anti-VEGF antibodies in recurrent/metastatic disease, but no combination antiangiogenic–immunotherapy regimen has yet received regulatory approval specifically in HNSCC [19,20,41]. Translating findings from renal or hepatocellular cancer to HNSCC requires caution, given the distinct immunological profiles, HPV-related molecular heterogeneity, and mucosal anatomical context of head and neck tumors. Future progress will depend on carefully designed clinical trials integrating immune, angiogenic, hypoxia-related, and stromal biomarkers to identify patients most likely to benefit from this combined strategy.

### 4.4. Emerging Therapeutic Approaches

Emerging therapeutic strategies targeting angiogenesis in HNSCC are moving beyond direct VEGF/VEGFR inhibition toward more integrated approaches that combine molecular targeting, RNA-based modulation, nanotechnology, immune remodeling, and personalized treatment selection. These strategies are particularly relevant because angiogenesis in HNSCC is not controlled by a single pathway, but by interconnected networks involving tumor cells, stromal cells, immune infiltrates, extracellular vesicles, hypoxia, and epigenetic regulation [17,20].

miRNA-based therapy is a promising experimental approach because several miRNAs regulate angiogenesis, EMT, invasion, cancer stemness, and therapeutic resistance. Two main strategies are currently considered: inhibition of oncogenic miRNAs using antagomiRs or antisense oligonucleotides, and restoration of tumor-suppressive miRNAs using miRNA mimics or replacement therapy [99]. In HNSCC, miRNA-based therapy may be especially useful for targeting multiple pathways simultaneously, including angiogenic signaling, immune escape, and resistance-associated programs. For example, exosome-mediated delivery of tumor-suppressive miRNAs such as miR-200c has been proposed as a potential strategy to suppress EMT and improve therapeutic efficacy [29]. However, clinical translation remains limited by instability of RNA molecules, off-target effects, immune activation, delivery barriers, and lack of validated patient-selection markers [100].

Nanoparticle delivery systems may help overcome some of these limitations by protecting therapeutic molecules from degradation, improving tumor targeting, and allowing controlled drug release. Lipid nanoparticles, polymeric nanoparticles, biomimetic vesicles, and exosome-based platforms can potentially deliver miRNA mimics, siRNAs, chemotherapeutic agents, immune modulators, or antiangiogenic compounds directly into the tumor microenvironment [20,101]. In addition, nanocarriers may be designed to respond to tumor-specific stimuli such as acidic pH, hypoxia, enzymatic activity, or oxidative stress, thereby increasing local drug concentration while reducing systemic toxicity. This is particularly relevant in HNSCC, where local tissue toxicity and post-radiotherapy changes often limit treatment tolerance. From a pharmaceutical formulation perspective, several critical aspects require consideration for clinical translation. Lipid nanoparticle formulations, which are the most clinically advanced nanocarrier class (exemplified by mRNA-LNP vaccines), can encapsulate nucleic acid payloads such as miRNA mimics with high efficiency but face challenges related to endosomal escape, preferential liver accumulation, and stability during long-term storage [28,101]. Polymeric nanoparticles, including PLGA- and chitosan-based systems, offer more tunable release kinetics and surface modification potential, but batch-to-batch reproducibility and scalable GMP-compliant manufacturing remain significant translational barriers [101]. Tumor-targeted strategies, such as conjugation of nanoparticles with EGFR-targeting ligands or peptides with affinity for tumor endothelium, may improve de-livery specificity in HNSCC given the high prevalence of EGFR overexpression in this tumor type; however, the specificity of active targeting in vivo is often lower than predicted by in vitro models [20,28]. Safety considerations include immunogenicity of nanocarrier components, off-target organ accumulation (particularly in liver and spleen), and potential genotoxicity of non-degradable particles. Regulatory requirements for nanoformulations are evolving, and early engagement with regulatory agencies regarding preclinical safety packages is recommended for translation into early-phase trials [101].

Gene therapy and RNA interference approaches also represent emerging directions for modulating angiogenic signaling. siRNA-based strategies can theoretically silence genes involved in VEGF signaling, hypoxia adaptation, stromal activation, or immune suppression. Similarly, gene-editing and gene-delivery platforms may be used to restore tumor-suppressive pathways or inhibit proangiogenic mediators [102]. Although these approaches remain largely preclinical in HNSCC, rapid advances in RNA therapeutics, vector engineering, and targeted delivery systems support their future translational potential. Their success will depend on improving tissue specificity, intracellular delivery, safety, and durability of response.

Combination therapy is likely to be more effective than single-agent antiangiogenic treatment because HNSCC frequently activates compensatory pathways. Rational combinations may include antiangiogenic agents with immune checkpoint inhibitors, EGFR inhibitors, radiotherapy, chemotherapy, epigenetic therapies, or RNA-based therapeutics [17,103]. The goal is not only to inhibit vessel formation, but also to normalize tumor vasculature, improve immune-cell infiltration, reduce hypoxia-driven resistance, and block parallel survival pathways. However, combination regimens require careful optimization, as excessive pathway inhibition may increase toxicity or paradoxically worsen hypoxia and immune exclusion.

Finally, personalized medicine is essential for the future development of antiangiogenic therapy in HNSCC. Molecular profiling, circulating biomarkers, miRNA signatures, exosomal cargo, hypoxia markers, immune checkpoint expression, and angiogenic cytokine panels may help identify patients most likely to benefit from specific combinations [17,72]. At present, the lack of standardized biomarkers remains a major limitation. Future trials should therefore integrate genomic, transcriptomic, epigenetic, immune, and vascular biomarkers into their design, allowing treatment selection based on tumor biology rather than uniform therapeutic algorithms.

Given the complexity and redundancy of angiogenic signaling in HNSCC, emerging therapeutic strategies increasingly focus on multimodal approaches that combine direct VEGF/VEGFR inhibition with immune modulation, RNA-based therapeutics, nanotechnology-assisted delivery, exosome engineering, vascular normalization, and biomarker-guided patient selection. The principal therapeutic strategies discussed in this section, together with their mechanisms, potential advantages, and current limitations, are summarized in Table 4.

Emerging therapeutic approaches in HNSCC angiogenesis should be understood as multimodal and biomarker-driven strategies. miRNA-based therapy, nanoparticle delivery, gene therapy, and rational combinations offer promising opportunities to overcome resistance and improve therapeutic precision. Nevertheless, their clinical implementation will require rigorous validation, standardized delivery systems, toxicity assessment, and prospective trials designed around molecularly defined patient subgroups.

## 5. Mechanisms of Therapeutic Resistance

Therapeutic resistance represents a major limitation of antiangiogenic and multimodal treatment strategies in HNSCC. Although inhibition of angiogenic signaling can transiently reduce vascular support and modify the tumor microenvironment, many tumors adapt by activating compensatory molecular programs. These adaptive responses involve alternative angiogenic pathways, stromal remodeling, inflammatory signaling, hypoxia-driven selection, immune suppression, and metabolic plasticity. As a result, resistance to antiangiogenic therapy should not be interpreted as a single-pathway failure, but as a dynamic process involving both tumor cells and the surrounding microenvironment [31,104].

### 5.1. Adaptive Resistance Pathways

Adaptive resistance occurs when tumor cells and stromal components reorganize their signaling networks in response to therapeutic pressure. In the context of angiogenesis inhibition, one of the most important mechanisms is bypass signaling, in which tumors reduce their dependence on VEGF/VEGFR signaling and activate parallel pathways capable of maintaining endothelial survival, vascular remodeling, and tumor progression. This explains why blockade of a single angiogenic axis may produce only temporary benefit in biologically heterogeneous tumors such as HNSCC [31,105].

A key example of bypass signaling is the upregulation of the FGF/FGFR pathway, particularly basic fibroblast growth factor. bFGF can sustain endothelial proliferation, migration, and survival even when VEGF signaling is inhibited. It may also promote stromal activation, tumor cell proliferation, and resistance to anti-VEGF strategies. Because of this compensatory role, FGF signaling is increasingly viewed as one of the most relevant alternative angiogenic pathways in acquired resistance to VEGF-targeted therapy [105,106].

Other alternative angiogenic pathways may also contribute to resistance, including PDGF/PDGFR, angiopoietin/Tie2, HGF/MET, EGF/EGFR, CXCL/CXCR chemokine signaling, and inflammatory cytokine networks. These pathways can support vascular maturation, pericyte recruitment, endothelial migration, and stromal remodeling. In addition, tumor-associated macrophages and other inflammatory cells may produce proangiogenic mediators independently of VEGF, thereby maintaining vascular support despite antiangiogenic therapy [107,108].

Paradoxically, excessive or sustained vascular inhibition may intensify rather than relieve tumor hypoxia. When antiangiogenic therapy eliminates too much of the vascular network, oxygen deprivation worsens, stabilizing HIF-1α-driven transcriptional programs that upregulate alternative growth factors, chemokines, and matrix-remodeling enzymes. This hypoxia-driven selection favors invasion, epithelial–mesenchymal transition, immune evasion, and emergence of more aggressive cellular subpopulations [88,104]. The therapeutic concept of vascular normalization—delivering antiangiogenic agents at doses sufficient to restructure, rather than ablate, the tumor vasculature—is discussed in the context of immunotherapy combinations in Section 4.3.

Adaptive resistance may also involve changes in the mode of tumor vascularization. Some tumors can rely less on classical sprouting angiogenesis and more on alternative vascular strategies such as vessel co-option, vasculogenic mimicry, or recruitment of bone marrow-derived proangiogenic cells. Although these mechanisms are best described in highly vascularized tumors such as glioblastoma, they illustrate a broader biological principle: tumors can preserve vascular access through non-VEGF-dependent mechanisms when conventional angiogenesis is therapeutically suppressed [109].

**Figure 3:**
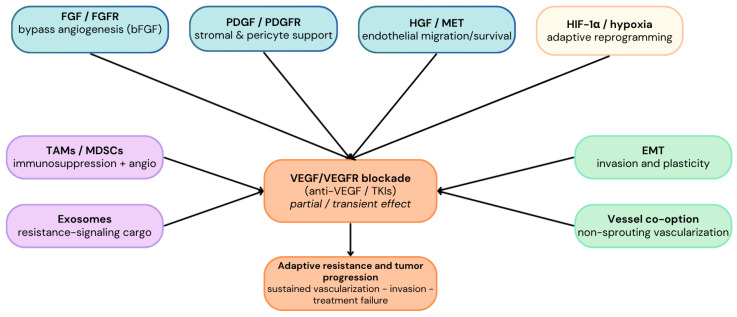
Principal mechanisms of resistance to antiangiogenic therapy in head and neck squamous cell carcinoma (HNSCC). Inhibition of the VEGF/VEGFR axis (by anti-VEGF antibodies or tyrosine kinase inhibitors) produces only a partial and often transient effect, because multiple bypass pathways can sustain tumor vascularization. Alternative angiogenic receptor tyrosine kinases (FGF/FGFR, PDGF/PDGFR, HGF/MET), hypoxia-driven HIF-1α reprogramming, immune and stromal mechanisms (TAMs/MDSCs, exosome-mediated signaling), and phenotypic or vascular plasticity (EMT, vessel co-option) converge to promote adaptive resistance, sustained vascularization, invasion, and treatment failure. Node colors group mechanisms by category, as indicated in the legend. Abbreviations: bFGF, basic fibroblast growth factor; EMT, epithelial–mesenchymal transition; FGF(R), fibroblast growth factor (receptor); HGF, hepatocyte growth factor; HIF-1α, hypoxia-inducible factor-1 alpha; MDSCs, myeloid-derived suppressor cells; MET, mesenchymal–epithelial transition factor receptor; PDGF(R), platelet-derived growth factor (receptor); RTKs, receptor tyrosine kinases; TAMs, tumor-associated macrophages; TKIs, tyrosine kinase inhibitors; VEGF(R), vascular endothelial growth factor (receptor). Legend, node colors: blue—alternative angiogenic RTKs; yellow—hypoxia; purple—immune/stromal; green—phenotypic/vascular plasticity.

Overall, adaptive resistance to antiangiogenic therapy in HNSCC likely results from the coexistence of multiple bypass pathways rather than from one dominant mechanism. This supports the need for combination strategies that target VEGF-dependent and VEGF-independent angiogenic signals, while also addressing hypoxia, inflammation, stromal support, and immune suppression. Future therapeutic development should therefore integrate dynamic biomarker monitoring to detect pathway switching and guide treatment adaptation before clinically evident progression occurs. To help readers integrate the mechanistic landscape described in this section, a schematic figure (Figure 3) summarizing the principal resistance pathways has been added. This figure illustrates the main adaptive escape mechanisms—including FGF/FGFR upregulation, PDGF/PDGFR-mediated stromal support, HGF/MET activation, HIF-1α-driven hypoxic reprogramming, TAM/MDSC-mediated immunosuppression, exosome-mediated resistance signaling, epithelial–mesenchymal transition, and vessel co-option—and their interconnections within the HNSCC tumor microenvironment.

### 5.2. Tumor Heterogeneity

Tumor heterogeneity is a major determinant of therapeutic resistance in HNSCC and helps explain why patients with apparently similar clinical stages may show markedly different responses to radiotherapy, chemotherapy, targeted therapy, immunotherapy, or antiangiogenic approaches. This heterogeneity occurs at multiple levels, including genetic alterations, epigenetic profiles, transcriptional programs, immune composition, stromal architecture, metabolic states, and vascular organization. Consequently, HNSCC should not be viewed as a single biological entity, but as a group of molecularly diverse tumors with distinct patterns of progression and treatment sensitivity [30,110].

Molecular heterogeneity is particularly relevant because HNSCC tumors differ according to HPV status, anatomical site, mutational landscape, copy-number alterations, gene-expression subtype, immune infiltration, and stromal activation. HPV-positive and HPV-negative tumors show distinct molecular and immune profiles, which influence prognosis and therapeutic response [63]. Beyond HPV status, transcriptomic studies have identified molecular subtypes with different levels of oncogenic signaling, immune infiltration, chemotherapy sensitivity, and immunotherapy responsiveness. For example, tumors with higher immune infiltration may have better prognosis and greater sensitivity to immune-based strategies, whereas tumors with activated oncogenic programs and reduced immune infiltration tend to show poorer outcomes [111].

Intratumoral heterogeneity further complicates treatment response. Within the same tumor, different cellular subclones may coexist, each with distinct proliferative capacity, invasive potential, metabolic adaptation, immune-interaction patterns, and therapeutic vulnerabilities. Under treatment pressure, sensitive clones may be eliminated while resistant populations survive and expand, leading to recurrence or disease progression. Recent studies using ctDNA and sequencing approaches suggest that circulating tumor DNA can reveal mutations missed by single tumor biopsies and may help detect minimal residual disease or emerging relapse earlier than conventional imaging [112,113].

The tumor microenvironment also contributes substantially to heterogeneity. Regional differences in hypoxia, stromal density, fibroblast activation, macrophage infiltration, immune checkpoint expression, and extracellular matrix composition create spatially distinct niches within the same tumor. These niches may influence drug penetration, immune-cell access, endothelial behavior, and local resistance to therapy [114]. For antiangiogenic therapy, this means that some tumor areas may remain dependent on VEGF-driven angiogenesis, while others may rely more on stromal support, inflammation, vessel co-option, or alternative vascularization mechanisms.

Therapeutic variability is therefore a direct consequence of biological diversity. A single biomarker measured from one tumor fragment may not accurately reflect the entire disease. This is especially relevant for PD-L1 assessment, where intra- and inter-sample heterogeneity can lead to clinically meaningful underestimation of expression and may affect immunotherapy selection [115]. Similarly, angiogenesis-related markers, immune signatures, and genomic alterations may vary between primary tumors, lymph node metastases, recurrences, and distant lesions.

Tumor heterogeneity represents both a challenge and an opportunity in HNSCC. It limits the effectiveness of uniform treatment strategies, but also provides a rationale for precision oncology approaches based on multi-region sampling, liquid biopsy, spatial profiling, digital pathology, and integrated multi-omics. Future therapeutic decisions should increasingly account for molecular and microenvironmental heterogeneity in order to improve patient stratification, identify resistant subclones earlier, and adapt treatment before clinical progression becomes evident.

### 5.3. Immunosuppressive Microenvironment

The immunosuppressive tumor microenvironment is an important mechanism of therapeutic resistance in HNSCC, particularly in patients receiving immune checkpoint inhibitors, radiotherapy, chemotherapy, or antiangiogenic combinations. In many tumors, antitumor immune activity is weakened by the accumulation of suppressive myeloid cells, dysfunctional lymphocytes, regulatory cytokines, hypoxia, and stromal barriers. These elements cooperate to reduce immune surveillance, limit cytotoxic T-cell activity, and promote tumor persistence despite treatment [60,110].

Myeloid-derived suppressor cells (MDSCs) are immature myeloid cells that expand during chronic inflammation and tumor progression. They suppress antitumor immunity through several mechanisms, including depletion of amino acids required for T-cell function, production of reactive oxygen and nitrogen species, secretion of immunosuppressive cytokines, promotion of regulatory T cells, and inhibition of dendritic-cell activity [116,117]. In HNSCC, enrichment of MDSCs has been associated with immune escape and poor response to immunotherapy, making these cells relevant therapeutic targets for overcoming resistance [118].

Tumor-associated macrophages, whose proangiogenic and stromal roles were described in Section 2.4, also contribute directly to therapeutic resistance by sustaining an immunosuppressive microenvironment. In the context of treatment resistance, the relevant feature is not their angiogenic output per se, but their capacity to impair cytotoxic T-cell function, support regulatory T-cell expansion, and maintain PD-L1-mediated suppression. In HPV-negative tumors, elevated CD163-positive macrophage infiltration has been associated with poorer prognosis, suggesting that macrophage polarization influences both immune response and sensitivity to systemic therapy [119].

T-cell exhaustion represents another central feature of the immunosuppressive microenvironment. Exhausted T cells show reduced proliferative capacity, impaired cytokine production, and diminished cytotoxic function, often accompanied by expression of inhibitory receptors such as PD-1, LAG-3, TIM-3, TIGIT, and CTLA-4. In HNSCC, the presence of exhausted CD8+ T-cell phenotypes may reflect chronic antigen stimulation and persistent immune pressure. While checkpoint expression can indicate an ongoing antitumor response, sustained exhaustion may limit the efficacy of immunotherapy if not accompanied by functional reinvigoration of effector T cells [119,120].

The interaction between MDSCs, TAMs, and exhausted T cells creates a self-sustaining suppressive network. MDSCs and TAMs inhibit cytotoxic lymphocytes and support regulatory T-cell expansion, while exhausted T cells become less capable of eliminating malignant cells. Exosomes derived from tumor cells or cancer-associated fibroblasts may further reinforce this state by suppressing T-cell receptor signaling, promoting regulatory T cells, increasing PD-L1 expression, and favoring immunosuppressive macrophage phenotypes [61]. This explains why targeting PD-1/PD-L1 alone may be insufficient in tumors with strong myeloid or stromal suppression.

The immunosuppressive microenvironment contributes to therapeutic resistance by preventing effective immune-mediated tumor clearance. In HNSCC, strategies that combine checkpoint blockade with approaches targeting MDSCs, TAMs, regulatory cytokines, exosomal signaling, or hypoxia-associated immune exclusion may improve response rates. Future studies should integrate spatial immune profiling, single-cell analysis, and dynamic biomarker monitoring to identify patients in whom immune suppression, rather than lack of antigenicity, is the dominant mechanism of resistance.

## 6. Translational and Clinical Perspectives

The translation of angiogenesis research into clinical practice requires reliable biomarkers able to predict prognosis, therapeutic response, and resistance. In HNSCC, this remains challenging because angiogenesis is influenced by tumor site, HPV status, hypoxia, immune infiltration, stromal composition, and previous treatment exposure. Therefore, single biomarkers are unlikely to fully capture the biological complexity of tumor vascularization. More useful approaches may involve integrated biomarker panels combining tissue-based markers, circulating proteins, liquid biopsy data, and non-coding RNA signatures [31,121].

### 6.1. Biomarkers for Therapeutic Response

VEGF expression is one of the most investigated angiogenesis-related biomarkers in HNSCC. Increased tissue or circulating VEGF levels have been associated with aggressive tumor behavior, advanced disease, poorer locoregional control, and unfavorable survival outcomes in several studies. However, VEGF expression is not yet a validated standalone predictive biomarker for selecting patients for antiangiogenic therapy. Its clinical interpretation is limited by variability in sample type, detection method, cut-off values, tumor subsite, and treatment context [39,121]. Interestingly, pretreatment plasma VEGF may have stronger prognostic value than serum VEGF, possibly because serum levels can be influenced by platelet-derived VEGF released during clotting. In one study, lower pretreatment plasma VEGF levels were associated with improved nodal, local, and locoregional control, as well as prolonged progression-free and event-free survival [39].

Circulating biomarkers are particularly attractive for therapeutic monitoring because they can be repeatedly assessed during treatment. Beyond VEGF, circulating cytokines, chemokines, angiogenic factors, immune-cell subsets, and ctDNA may provide information about tumor burden, residual disease, immune activation, and emerging resistance. Liquid biopsy approaches are especially relevant in HNSCC because spatial heterogeneity can limit the representativeness of a single tissue biopsy. Recent studies suggest that peripheral immune profiling and plasma protein panels may help predict response to neoadjuvant PD-1 blockade, while ctDNA-based monitoring may detect minimal residual disease and molecular relapse earlier than standard imaging [113,122]. These tools remain investigational, but they support a transition from static pretreatment biomarkers toward dynamic response monitoring.

miRNA signatures also show translational potential as minimally invasive biomarkers. Circulating miRNAs are stable in plasma, serum, saliva, and extracellular vesicles, making them suitable candidates for diagnosis, prognosis, and therapeutic-response assessment. In HNSCC patients undergoing radiotherapy, circulating miRNA profiles have been associated with prognosis and treatment monitoring, with miR-142-3p, miR-186-5p, miR-195-5p, miR-374b-5p, and miR-574-3p reported as promising plasma markers [22]. Other studies have identified diagnostic or prognostic miRNA panels, including three-miRNA signatures and recurrent candidates such as miR-21, miR-155, and miR-375 [75,123].

Among these, miR-21 is particularly relevant because high expression has been associated with poor response to organ-preservation protocols and worse survival in locally advanced HNSCC [124]. However, miRNA-based biomarkers face important limitations, including differences in normalization strategies, biological fluid type, extraction protocols, sequencing or PCR platforms, and patient heterogeneity. For this reason, miRNA signatures should be validated in large prospective cohorts before being implemented in routine therapeutic decision-making.

Biomarkers for therapeutic response in HNSCC angiogenesis should be interpreted as complementary rather than interchangeable. VEGF expression may reflect angiogenic activity, circulating biomarkers may enable real-time monitoring, and miRNA signatures may capture post-transcriptional regulation and tumor–microenvironment communication. Future clinical trials should incorporate biomarker-driven stratification and longitudinal sampling to identify patients most likely to benefit from antiangiogenic, immunotherapeutic, or combination strategies. To facilitate a practical translational overview, the main angiogenesis-related biomarkers discussed in this section are summarized in Table 5, including biomarker type, sample type, proposed detection method, proposed clinical use, current level of evidence, and main limitations. This summary aims to provide a structured reference for investigators designing future biomarker-driven trials in HNSCC.

### 6.2. Precision Oncology in HNSCC

Precision oncology in HNSCC aims to move beyond treatment decisions based only on tumor site, TNM stage, and histopathological features, toward a more individualized strategy guided by tumor biology. This approach is particularly important because HNSCC includes biologically diverse tumors with different etiologies, molecular alterations, immune profiles, stromal characteristics, and patterns of therapeutic response. In this context, patient stratification and molecular profiling are essential for identifying patients who may benefit from targeted therapy, immunotherapy, antiangiogenic strategies, or rational combination regimens [113,125].

Patient stratification currently relies on several clinically established factors, including HPV/p16 status, tumor subsite, disease stage, performance status, smoking history, PD-L1 combined positive score, and prior treatment exposure. Among these, HPV/p16 status remains one of the most validated biomarkers in HNSCC, particularly in oropharyngeal squamous cell carcinoma, where it has strong prognostic relevance [125]. However, these parameters are not sufficient to predict therapeutic response in all patients. Additional stratification tools are needed to account for immune infiltration, hypoxia, angiogenic activity, stromal composition, and intratumoral heterogeneity [114,126].

Molecular profiling can identify genomic, transcriptomic, proteomic, and epigenetic alterations that may influence prognosis and treatment sensitivity. Multi-platform profiling studies have shown that HNSCC frequently displays EGFR overexpression, TP53 mutations, PIK3CA alterations, PTEN loss, and activation of immune checkpoint pathways across different tumor sites and HPV categories [127]. Although many of these alterations are biologically meaningful, only a limited number have been translated into routine targeted treatment decisions. This gap reflects the complexity of HNSCC biology, the coexistence of multiple resistance pathways, and the lack of prospectively validated predictive biomarkers.

Immune and spatial profiling are becoming increasingly relevant for precision oncology. Analyses of the tumor microenvironment have identified immune phenotypes such as cytotoxic, plasma-cell-rich, dendritic-cell-rich, macrophage-rich, and immune-excluded tumors, each potentially associated with different immunotherapy sensitivity [128]. Spatial profiling may further improve patient selection by distinguishing biomarker expression in tumor cells, immune cells, and stromal compartments. For example, compartmentalized analyses of immune checkpoint molecules and TNF receptor superfamily members have shown potential for identifying patients more likely to respond to immunotherapy, although prospective validation is still required [129].

Liquid biopsy may also support precision oncology by enabling non-invasive and longitudinal monitoring. Circulating tumor DNA profiling can detect tumor-specific mutations, methylation changes, minimal residual disease, molecular relapse, and possible resistance evolution [113,130]. This is particularly valuable in HNSCC, where single tissue biopsies may not fully capture spatial and temporal tumor heterogeneity. Repeated blood-based profiling may help identify recurrence earlier, adapt therapy dynamically, and reduce dependence on invasive sampling.

Despite these advances, precision oncology in HNSCC remains limited by several challenges. Many proposed biomarkers lack standardized assays, validated cut-off values, and prospective clinical confirmation. Moreover, tumor heterogeneity means that a single molecular alteration may not be sufficient to guide therapy. Future clinical strategies should therefore integrate multiple data layers, including genomic alterations, transcriptomic subtypes, immune markers, angiogenic signatures, hypoxia-related biomarkers, imaging features, and liquid biopsy data. Such integrated profiling may improve patient stratification and help identify rational combinations rather than relying on single-agent targeted approaches.

Precision oncology in HNSCC is moving toward a multidimensional model of patient selection. Molecular profiling and patient stratification have the potential to improve therapeutic decisions, but their clinical utility will depend on robust validation, standardized testing, and integration into prospective biomarker-driven trials.

### 6.3. Future Clinical Trials

Future clinical trials in HNSCC should move from broadly applied treatment combinations toward biologically informed, biomarker-driven trial designs. The limited benefit observed with several targeted or antiangiogenic agents in unselected populations suggests that future studies should incorporate molecular profiling, immune characterization, angiogenic markers, hypoxia assessment, and longitudinal liquid biopsy monitoring from the beginning of trial design, rather than as secondary exploratory analyses [19,31].

Combination therapies will remain a central direction in HNSCC research. Because resistance often results from parallel signaling pathways and microenvironmental adaptation, single-agent strategies are unlikely to be sufficient for many patients. Future trials should evaluate rational combinations such as immune checkpoint inhibitors with antiangiogenic agents, EGFR inhibitors, radiotherapy, chemotherapy, hypoxia-modifying agents, or stromal-targeted therapies. However, these combinations must be carefully optimized, because increased therapeutic intensity may also increase toxicity, particularly in patients previously treated with surgery or radiotherapy [19,131].

Personalized therapies require improved patient selection. Instead of enrolling heterogeneous patient populations based only on anatomical site and stage, future studies should stratify patients according to HPV status, PD-L1 expression, immune phenotype, angiogenic profile, genomic alterations, transcriptomic subtype, and markers of hypoxia or stromal activation. This approach may help identify subgroups that benefit from specific therapeutic combinations while sparing non-responders from unnecessary toxicity [17,31]. Patient-derived organoids, 3D tumor models, and xenograft systems may also support preclinical testing of individualized therapeutic strategies before clinical implementation [132].

Artificial intelligence and predictive modeling are expected to become increasingly important in future clinical trials. AI-based models can integrate complex datasets, including clinical variables, radiomics, digital pathology, genomics, transcriptomics, immune biomarkers, and treatment outcomes. In HNSCC, multimodal deep-learning models have already shown potential for predicting prognosis and postoperative radiotherapy response, suggesting that integrated computational tools may support individualized therapeutic planning [133]. Similarly, machine-learning-derived immune gene signatures may improve risk stratification and predict sensitivity to immunotherapy or other systemic agents [134]. In the specific context of antiangiogenic and combination therapy decisions in HNSCC, AI-based tools may contribute in several ways: (1) integrating multi-omic angiogenic signatures, hypoxia imaging data, and immune biomarkers to identify patients most likely to benefit from VEGF/VEGFR-targeted agents or vascular normalization strategies; (2) analyzing radiomics features from CT or MRI to non-invasively estimate intratumoral hypoxia or vascular density, which may guide bevacizumab or TKI selection; (3) processing longitudinal liquid biopsy data to detect early adaptive resistance and trigger treatment modification before clinical progression; and (4) identifying rational combination strategies through network pharmacology models applied to tumor microenvironment datasets [133,134,135]. These specific applications remain at an early validation stage, and prospective integration within interventional antiangiogenic trials represents an important next step.

However, AI-driven predictive models must be validated rigorously before they can guide clinical decisions. Many current AI studies are retrospective, single-institutional, or dependent on heterogeneous datasets, and few models have been prospectively tested within interventional trials. Future trial designs should therefore include predefined AI endpoints, external validation cohorts, transparent reporting, model interpretability, and evaluation of clinical utility. Ethical and regulatory issues, including data privacy, algorithmic bias, reproducibility, and generalizability across populations, must also be addressed [135,136].

Future HNSCC clinical trials should be adaptive, biomarker-guided, and multidisciplinary. The most promising direction is likely to involve rational therapeutic combinations selected according to tumor biology and supported by predictive models capable of integrating molecular, imaging, pathological, and clinical data. This approach may improve treatment personalization, reduce unnecessary toxicity, and accelerate the translation of angiogenesis-related research into clinically meaningful benefits.

## 7. Future Directions and Research Gaps

Despite substantial progress in understanding tumor angiogenesis in HNSCC, the clinical impact of antiangiogenic strategies remains more limited than initially expected. The main challenge is not the absence of biological rationale, but the difficulty of translating complex molecular and microenvironmental mechanisms into reproducible clinical benefit. Future research must therefore move beyond descriptive biomarker studies and focus on standardized, clinically actionable, and prospectively validated approaches [31,137].

One of the most important gaps is the lack of standardized biomarkers. Numerous angiogenesis-related markers, including VEGF-family members, VEGFRs, hypoxia markers, microRNAs, exosomal cargo, immune markers, and stromal signatures, have been associated with prognosis or treatment response. However, few have been validated as predictive tools for selecting patients for antiangiogenic or combination therapy. Differences in sample type, timing of collection, assay platforms, scoring systems, cut-off values, tumor subsite, HPV status, and treatment background make comparisons between studies difficult [31,138]. Therefore, future trials should include predefined biomarker endpoints, harmonized laboratory protocols, and external validation cohorts.

A second major limitation is the difficulty of translating preclinical results into clinical practice. Many antiangiogenic or TME-targeted strategies show promising effects in cell lines, xenografts, organoids, or animal models, but fail to produce durable benefit in patients. This discrepancy reflects the limited ability of many preclinical models to reproduce the anatomical complexity, immune contexture, stromal heterogeneity, vascular architecture, hypoxia gradients, and prior-treatment effects found in human HNSCC [132,139]. More representative models, including patient-derived organoids, 3D co-culture systems, cancer-on-a-chip platforms, humanized models, and spatially resolved multi-omics, may improve translational accuracy, but these tools also require standardization and clinical correlation before becoming routine research platforms [132,140].

The heterogeneity of the tumor microenvironment remains another critical obstacle. HNSCC tumors differ markedly in immune infiltration, fibroblast activation, macrophage polarization, endothelial phenotype, extracellular matrix composition, hypoxia, and cytokine signaling. Even within the same patient, primary tumors, lymph node metastases, recurrences, and residual disease may display different biological states. This heterogeneity limits the effectiveness of uniform therapeutic strategies and complicates the interpretation of biomarkers measured from a single biopsy [137,141]. Future research should therefore prioritize spatial profiling, single-cell technologies, liquid biopsy, and longitudinal sampling to capture dynamic tumor–microenvironment interactions.

Toxicity is a particularly important limitation in head and neck cancer. Antiangiogenic agents and multi-target TKIs may cause hypertension, bleeding, thromboembolic events, impaired wound healing, fistula formation, mucosal injury, fatigue, diarrhea, proteinuria, and cardiovascular complications. These risks are clinically relevant because many patients have undergone surgery, radiotherapy, or chemoradiotherapy and may already have fragile mucosal, vascular, and nutritional status [31,142]. Future trials must not only evaluate survival endpoints, but also quality of life, functional outcomes, swallowing, speech, wound complications, and long-term toxicity.

Cost and accessibility also deserve more attention. Precision oncology, molecular profiling, immunotherapy, antiangiogenic agents, nanomedicine, exosome-based diagnostics, and AI-driven platforms may improve individualization of care, but they also increase financial burden and technological complexity. If these approaches are not accompanied by cost-effectiveness analyses and implementation strategies, they may widen disparities between high-resource and low-resource healthcare systems [37]. Future research should therefore include health-economic evaluation, simplified biomarker panels, and clinically feasible algorithms that can be adopted outside highly specialized centers.

The next phase of HNSCC angiogenesis research should be more selective, integrative, and clinically disciplined. The field needs fewer isolated biomarker associations and more prospective trials that combine standardized biomarkers, rational therapeutic combinations, dynamic monitoring, toxicity assessment, and cost-effectiveness analysis. Only through this approach can angiogenesis-targeted strategies move from biological promise to meaningful clinical implementation.

## 8. Conclusions

Antiangiogenic therapy currently occupies a limited and non-standard role in the clinical management of HNSCC. Despite strong biological rationale and decades of investigation, no antiangiogenic agent has achieved regulatory approval specifically for this tumor type, and the modest clinical outcomes observed in multiple trials reflect the biological complexity and molecular heterogeneity of HNSCC rather than a failure of the angiogenic concept itself. The primary reasons for limited clinical translation include the absence of validated predictive biomarkers for patient selection, the redundancy of proangiogenic signaling pathways that enable bypass resistance, and the significant toxicity burden in a patient population already compromised by prior surgery, radiotherapy, and mucosal disease. Tumor angiogenesis in HNSCC is not merely a mechanism of vascular supply, but an integrated biological program linking hypoxia, metabolic adaptation, stromal remodeling, immune evasion, and therapeutic resistance.

Meaningful clinical progress will require a transition from broad, biomarker-agnostic antiangiogenic strategies toward prospectively validated, biomarker-guided approaches that integrate genomic, immune, and vascular profiling into trial design. Rational combinations with immune checkpoint inhibitors, EGFR inhibitors, and radiotherapy offer the most biologically credible pathway forward, provided that the optimal agents, doses, sequencing, and patient selection criteria are defined in adequately powered prospective studies. Novel delivery platforms, including nanoparticle systems and RNA-based therapeutics, expand the pharmacological repertoire but require rigorous preclinical validation and early-phase clinical testing before their translational potential can be assessed. Ultimately, closing the gap between biological promise and clinical benefit in HNSCC angio-genesis research will demand higher standards of biomarker integration, trial design, and translational rigor than have characterized this field to date.

## Figures and Tables

**Table 1:** Major angiogenic signaling pathways in HNSCC.

Pathway	Principal Molecular Mediators	Biological Effects in HNSCC	Current Therapeutic Relevance in HNSCC
VEGF/VEGFR [10,17,31,38,41,43]	VEGF-A, VEGFR-1 (Flt-1), VEGFR-2 (KDR/Flk-1)	Endothelial proliferation, migration, vascular permeability, neovessel formation; association with aggressive behavior and adverse prognosis	Best-developed antiangiogenic axis in HNSCC; anti-VEGF/VEGFR strategies and rational combination regimens
HIF-1α/hypoxia [9,31,35]	HIF-1α, GLUT-1, CA IX	Hypoxic adaptation, glycolytic/metabolic reprogramming, angiogenic gene activation, treatment resistance	Hypoxia-directed and biomarker-guided approaches remain investigational
PI3K/AKT/mTOR [33,45,46,47]	PI3K, AKT, mTOR, PTEN, PIK3CA	Cell survival, proliferation, angiogenesis, EMT, radioresistance and systemic-therapy resistance	Major combination and biomarker-guided target; PI3K/mTOR inhibitors remain investigational in HNSCC
MAPK/ERK [33,40,45]	RAS, RAF, MEK, ERK	Endothelial proliferation and migration, invasion, EMT, resistance signaling	Mainly preclinical/investigational; relevant as a VEGF–EGFR crosstalk node
EGFR-associated signaling [4,40,45,47]	EGFR, EGF, TGF-α	Tumor-cell proliferation, VEGF upregulation, angiogenic crosstalk, HPV-related heterogeneity	Established targeted-therapy platform in HNSCC; rational partner for antiangiogenic or PI3K-axis combinations
FGF/FGFR [17,31,34,36]	FGF-2 (bFGF), FGFR	Neovascularization, endothelial survival, EMT-linked progression, potential VEGF-independent escape	Alternative/bypass angiogenic pathway; indirect relevance to multi-target antiangiogenic TKIs
Angiopoietin/Tie2 [17,31,34,36]	Angiopoietins, especially Ang-2; Tie2	Vessel remodeling, destabilization/stabilization, EMT-linked vascular progression	Alternative angiogenic pathway; investigational at this stage of the manuscript
PDGF/PDGFR [17,31,36]	PDGF family, PDGFR	Stromal–vascular support, vascular maturation, adaptive angiogenic amplification	Mostly indirect relevance through broader antiangiogenic multi-target strategies
Inflammatory cytokine axis [35,37,42,44]	IL-8, IL-6, VEGF-associated inflammatory mediators	Proangiogenic amplification, aggressive clinical behavior, treatment-response/prognostic associations, immune suppression	Investigational; supports rationale for combination therapy
Chronic inflammatory/TME signaling [12,31,42]	Tumor- and stroma-derived inflammatory mediators, CAF/TAM-associated signaling	Sustained cytokine production, angiogenic switch, stromal activation, immunosuppressive tumor microenvironment	Investigational; supports microenvironment-directed combination strategies

**Table 2:** Representative microRNAs involved in angiogenesis-related regulation and translational relevance in HNSCC. Abbreviations: EMT, epithelial–mesenchymal transition; HNSCC, head and neck squamous cell carcinoma; miRNA, microRNA; NF-κB, nuclear factor kappa B; PI3K/AKT, phosphoinositide 3-kinase/protein kinase B; VEGF, vascular endothelial growth factor.

microRNA	Main Biological Role in HNSCC	Angiogenesis-Related Mechanism	Translational Relevance
miR-21	Oncogenic miRNA; associated with proliferation, EMT, invasion and therapy resistance [72,73,74,75]	May promote VEGF-associated and PI3K/AKT-related signaling; can be transferred through exosomes [62,74]	Prognostic biomarker; potential therapeutic target using antagomiRs [75]
miR-155	Inflammation-associated miRNA involved in immune modulation and tumor progression [75,76]	Indirectly supports a proangiogenic microenvironment through inflammatory cytokine networks [75,76]	Candidate immune-related biomarker [75,76]
miR-146a	Context-dependent regulator of NF-κB-associated inflammatory signaling [73,76]	May modulate angiogenesis indirectly through cytokine regulation and stromal–immune interactions [73,76]	Potential biomarker of inflammatory tumor behavior [73,76]
miR-221/222	Associated with cell-cycle progression, EMT, invasion and drug resistance [77]	May support invasive and therapy-resistant phenotypes linked to angiogenic adaptation [77]	Candidate marker of aggressive and resistant disease [77]
miR-200c	Tumor-suppressive miRNA involved in EMT inhibition [29]	May reduce EMT-associated invasion and indirectly limit proangiogenic remodeling [29]	Potential candidate for exosome-mediated therapeutic delivery [29]
miR-34a	Tumor-suppressive miRNA involved in proliferation control [23]	Reported to influence tumor growth and angiogenic activity [23]	Candidate replacement-therapy target [23,28]
miR-203	Tumor-suppressive miRNA associated with invasion and EMT regulation [24]	May reduce invasive reprogramming associated with angiogenic progression [24]	Potential prognostic and therapeutic biomarker [24,72,73]

**Table 3:** Principal clinical trials of antiangiogenic agents in head and neck squamous cell carcinoma (HNSCC). Abbreviations: CPS, combined positive score; EGFR, epidermal growth factor receptor; FGFR, fibroblast growth factor receptor; HR, hazard ratio; mo, months; NS, not significant; ORR, objective response rate; OS, overall survival; PDGFR, platelet-derived growth factor receptor; PD-1, programmed cell death protein 1; PD-L1, programmed death-ligand 1; PFS, progression-free survival; PR, partial response; R/M, recurrent/metastatic; TRAEs, treatment-related adverse events; VEGF(R), vascular endothelial growth factor (receptor).

Agent (Trial)	Target	Phase/Setting	Combination	Key Efficacy Outcomes	Main Toxicities	Conclusion/Status
Bevacizumab [17]	VEGF-A	Phase III; first-line R/M HNSCC	Platinum-based doublet ± bevacizumab (*n* = 403)	Median OS 12.6 vs. 11.0 mo (HR 0.87; *p* = 0.22); ORR 35.5% vs. 24.5%; median PFS 6.0 vs. 4.3 mo (*p* = 0.0014)	Bleeding, treatment-related deaths, hypertension	No significant OS benefit; not adopted as standard
Bevacizumab + erlotinib (phase I/II) [14]	VEGF-A + EGFR	Phase I/II; R/M HNSCC	Bevacizumab + erlotinib	Modest response rates; signal of activity in small cohorts	Rash, bleeding, diarrhea	Exploratory; insufficient to define a standard regimen
Sorafenib [17,81]	RAF, VEGFR, PDGFR	Phase II; R/M HNSCC	Single agent	ORR < 5%; median PFS ~2–4 mo	Hand–foot reaction, fatigue, hypertension	Insufficient single-agent activity
Sunitinib [17,18]	VEGFR, PDGFR, c-KIT, FLT3	Phase II; R/M HNSCC (37.5 mg/day)	Single agent	PR ~2–13%; median PFS ~2–4 mo	Oral mucositis, bleeding, fatigue	Limited activity; further single-agent development not supported
Pazopanib [18,82]	VEGFR, PDGFR, c-KIT	Early-phase; R/M HNSCC	Single agent/combinations	Limited HNSCC-specific data; activity by analogy to RCC/sarcoma	Hypertension, hepatotoxicity, diarrhea	Investigational in HNSCC
Lenvatinib + pembrolizumab [17,18]	VEGFR1–3, FGFR1–4, PDGFRα + PD-1	Phase III; first-line PD-L1 CPS ≥ 1 R/M HNSCC (*n* = 511)	Lenvatinib + pembrolizumab vs. placebo + pembrolizumab	ORR 46.1% vs. 25.4% (*p* < 0.001); median PFS 6.2 vs. 2.8 mo (HR 0.64); median OS 15.0 vs. 17.9 mo (HR 1.15; NS)	Increased TRAEs (hypertension, diarrhea, fatigue)	Improved ORR/PFS but no OS benefit; did not meet primary endpoint
Lenvatinib + pembrolizumab (phase Ib/II; NCT02501096) [18]	Multikinase + PD-1	Phase Ib/II; R/M HNSCC	Lenvatinib + pembrolizumab	ORR ~46% in early cohort	Hypertension, fatigue, proteinuria	Promising early signal that informed LEAP-010
Anti-PD-1 + anti-VEGF antibody [41]	PD-1 + VEGF	Retrospective; second-line + R/M HNSCC	Anti-PD-1 mAb + anti-VEGF agent	Reported as safe and effective in selected pretreated patients	Manageable immune-related and antiangiogenic toxicities	Hypothesis-generating; requires prospective confirmation

**Table 4:** Emerging and combination-based therapeutic strategies targeting angiogenesis-related mechanisms in HNSCC. Abbreviations: FGFR, fibroblast growth factor receptor; HNSCC, head and neck squamous cell carcinoma; KIT, KIT proto-oncogene receptor tyrosine kinase; miRNA, microRNA; PDGFR, platelet-derived growth factor receptor; RET, rearranged during transfection receptor tyrosine kinase; siRNA, small interfering RNA; VEGF, vascular endothelial growth factor; VEGFR, vascular endothelial growth factor receptor.

Therapeutic Strategy	Main Target/Mechanism	Potential Advantage	Main Limitations in HNSCC
Anti-VEGF monoclonal antibodies	Neutralization of VEGF-A and inhibition of VEGFR activation [17,83]	Direct inhibition of the dominant angiogenic pathway [17,31]	Inconsistent clinical benefit; bleeding, wound-healing complications, toxicity [17,83]
VEGFR-targeted monoclonal antibodies	Blockade of VEGFR-2 signaling [86,87]	Direct receptor-level inhibition of VEGF-mediated angiogenic signaling [86,87]	Limited direct evidence in HNSCC; mostly investigational [17,86,87]
Multi-target tyrosine kinase inhibitors	VEGFR, FGFR, PDGFR, RET, KIT and related kinases [18,89]	Broader inhibition of redundant angiogenic and stromal pathways [17,18]	Off-target toxicity; lack of validated predictive biomarkers [89,90,93]
Antiangiogenic therapy + immunotherapy	Vascular normalization and reduction in VEGF-mediated immunosuppression [96,97,98]	May improve immune-cell infiltration and response to checkpoint inhibitors [20,96,98]	Optimal dose, timing and patient selection remain unclear; combined toxicity may occur [19,98]
miRNA-based therapy	Inhibition of oncogenic miRNAs or restoration of tumor-suppressive miRNAs [22,99,100]	Ability to modulate multiple angiogenic, invasive and resistance-related pathways simultaneously [22,99]	Delivery barriers, instability, immune activation and off-target effects [99,100]
Nanoparticle-based delivery	Targeted delivery of miRNAs, siRNAs, drugs or immune modulators [20,28,101,102]	Improved tumor targeting, cargo protection and reduced systemic toxicity [28,101]	Mostly preclinical; safety, reproducibility and clinical validation remain required [100,101,102]
Exosome-mediated therapy	Engineered vesicles delivering tumor-suppressive or immunomodulatory cargo [29,61,74,78]	Natural biological carriers with potential tissue tropism and cargo protection [29,78]	Exosome heterogeneity, scalability, cargo loading, biodistribution and safety issues [29,78]
Vascular normalization strategies	Functional remodeling rather than complete vessel ablation [16,96,98]	May improve oxygenation, drug delivery and immune-cell infiltration [16,95,98]	Narrow therapeutic window; difficult clinical monitoring and dose optimization [16,98]
Biomarker-guided combinations	Selection based on VEGF, hypoxia, immune, circulating or miRNA profiles [19,72,93,103]	More personalized therapeutic approach and improved patient stratification [17,19,72]	Lack of standardized biomarkers, validated cut-offs and prospective confirmation [72,93]

**Table 5:** Angiogenesis-related biomarkers in HNSCC and their translational characteristics. Abbreviations: CA IX, carbonic anhydrase IX; CPS, combined positive score; ctDNA, circulating tumor DNA; ELISA, enzyme-linked immunosorbent assay; GLUT-1, glucose transporter 1; HIF-1α, hypoxia-inducible factor-1 alpha; HPV, human papillomavirus; miRNA, microRNA; PD-L1, programmed death-ligand 1; qRT-PCR, quantitative reverse-transcription polymerase chain reaction; TAMs, tumor-associated macrophages; VEGF(R), vascular endothelial growth factor (receptor).

Biomarker	Type	Sample Type	Detection Method	Proposed Clinical Use	Level of Evidence	Main Limitation
VEGF (tissue)	Angiogenic protein	Tumor tissue	Immunohistochemistry	Prognosis; angiogenic activity	Moderate (meta-analyses) [43]	Variable cut-offs; not a validated predictive marker
VEGF (plasma)	Circulating protein	Plasma	ELISA	Prognosis; treatment monitoring	Moderate [39]	Influenced by platelet-derived VEGF; assay variability
VEGFR-2	Receptor tyrosine kinase	Tumor tissue	Immunohistochemistry	Target engagement; prognosis	Low–moderate [31]	Limited HNSCC-specific predictive data
HIF-1α/CA IX/GLUT-1	Hypoxia markers	Tumor tissue	Immunohistochemistry	Hypoxia status; radiotherapy response	Moderate [35,51]	Site-dependent prognostic value; heterogeneity
miR-21	Oncogenic miRNA	Tissue, plasma, saliva, exosomes	qRT-PCR	Prognosis; organ-preservation response	Moderate [75,124]	Normalization and platform variability
miRNA panels (e.g., miR-142-3p, miR-186-5p, miR-195-5p)	Circulating miRNA signatures	Plasma	qRT-PCR/sequencing	Prognosis; treatment monitoring	Low–moderate [22,123]	Require large prospective validation
Exosomal miRNAs	Vesicular RNA cargo	Saliva, serum	Exosome isolation + qRT-PCR	Early detection; liquid biopsy	Low [79,80]	No standardized isolation; small cohorts
ctDNA	Circulating tumor DNA	Plasma	Targeted/genome sequencing	Minimal residual disease; molecular relapse	Emerging [113,122]	Sensitivity at low tumor burden; cost
PD-L1 (CPS)	Immune checkpoint ligand	Tumor tissue	Immunohistochemistry	Immunotherapy selection	Established but imperfect [94,115]	Intra-/inter-sample heterogeneity
CD163+ TAMs	Stromal/immune marker	Tumor tissue	Immunohistochemistry	Prognosis (esp. HPV-negative)	Low–moderate [119]	Lack of standardized scoring

## Data Availability

The original contributions presented in this study are included in the article. Further inquiries can be directed to the corresponding author.

## Abbreviations

The following abbreviations are used in this manuscript:

HNSCC	Head and Neck Squamous Cell Carcinoma
VEGF	Vascular Endothelial Growth Factor
VEGFR	Vascular Endothelial Growth Factor Receptor
HIF-1α	Hypoxia-Inducible Factor 1 Alpha
TME	Tumor Microenvironment
EMT	Epithelial–Mesenchymal Transition
miRNA	MicroRNA
CAF	Cancer-Associated Fibroblast
TAM	Tumor-Associated Macrophage
MDSC	Myeloid-Derived Suppressor Cell
ECM	Extracellular Matrix
PDGF	Platelet-Derived Growth Factor
FGF	Fibroblast Growth Factor
FGFR	Fibroblast Growth Factor Receptor
PI3K	Phosphoinositide 3-Kinase
AKT	Protein Kinase B
mTOR	Mammalian Target of Rapamycin
MAPK	Mitogen-Activated Protein Kinase
ERK	Extracellular Signal-Regulated Kinase
TKI	Tyrosine Kinase Inhibitor
PD-1	Programmed Cell Death Protein 1
PD-L1	Programmed Death-Ligand 1
CTLA-4	Cytotoxic T-Lymphocyte-Associated Protein 4
VEGF-A	Vascular Endothelial Growth Factor A
VEGFR-2	Vascular Endothelial Growth Factor Receptor 2
GLUT-1	Glucose Transporter 1
MMP	Matrix Metalloproteinase
RNA	Ribonucleic Acid
DNA	Deoxyribonucleic Acid
HPV	Human Papillomavirus
EGFR	Epidermal Growth Factor Receptor
NF-κB	Nuclear Factor Kappa B
CA IX	Carbonic Anhydrase IX
Ang	Angiopoietin
Tie2	Tyrosine Kinase with Immunoglobulin-like and EGF-like Domains 2
VEGF165b	Vascular Endothelial Growth Factor 165b
PlGF	Placental Growth Factor
FDA	Food and Drug Administration
OS	Overall Survival
PFS	Progression-Free Survival
ROS	Reactive Oxygen Species
CSC	Cancer Stem Cell
siRNA	Small Interfering RNA
ATP	Adenosine Triphosphate
